# Microbial biopriming of germinated barley enhances phenolics and B-vitamins and improves neurobehavior in MSG-treated rats

**DOI:** 10.1038/s41538-026-01022-z

**Published:** 2026-07-29

**Authors:** Ahmed Y. Salem, Jilan A. Nazeam, Manal M. Sabry, Magdy M. Awny, Lina Jamil M. Abdelhafez, Soad Z. El-Emam, Sabah H. El-Gayed

**Affiliations:** 1 Pharmacognosy Department, Faculty of Pharmacy, October 6 University, Giza, Egypt; 2 Pharmacognosy Department, Faculty of Pharmacy, Cairo University, Giza, Egypt; 3 Pharmacology and Toxicology Department, Faculty of Pharmacy, October 6 University, Giza, Egypt; 4 Microbiology Department, Faculty of Pharmacy, October 6 University, Giza, Egypt

**Keywords:** Biochemistry, Biological techniques, Biotechnology, Microbiology, Plant sciences

## Abstract

Biopriming is a seed-level biotechnological intervention with considerable potential to enhance the nutritional, phytochemical, and functional attributes of cereal grains. In this study, microbial biopriming was investigated as a targeted approach to modulate the phytochemical composition, antioxidant capacity, and neuroprotective efficacy of barley-derived extracts. Germinated barley extract (GBE) was comparatively evaluated against two bioprimed derivatives produced by inoculating barley seeds with *Bacillus subtilis* (GBB) and *Aspergillus niger* (GBF) prior to germination. Comprehensive physicochemical characterization was performed using chromatographic and spectroscopic profiling, and functional efficacy was evaluated through in vitro antioxidant assays and in vivo assessment using a monosodium glutamate (MSG)-induced attention-deficit/hyperactivity disorder-like rat model. The GBB exhibited the most pronounced bioactivity, characterized by marked enrichment in phenolic acids and B-complex vitamins, particularly vitamins B2, B9, and B12. This enhanced compositional profile was associated with significant improvements in locomotor activity and spatial memory, restoration of neurotransmitter homeostasis, attenuation of oxidative stress and neuroinflammatory signaling, and activation of autophagy-associated survival pathways. This study identifies *B. subtilis* biopriming as a promising and sustainable method for amplifying the neuroprotective properties of germinated barley, advocating its development as a functional food ingredient derived from cereals.

## Introduction

Barley (*Hordeum vulgare* L.) is the fourth most widely cultivated cereal worldwide, with an annual production of approximately 140 million tons, and is increasingly recognized as a nutrient-dense grain with considerable potential for functional food development^1–3^. Compared with several commonly consumed cereals, barley is distinguished by its relatively high β-glucan and soluble dietary fiber content, which are closely associated with metabolic regulation and functional food applications^4^. The potential for functional enhancement is further substantiated by its pronounced responsiveness to germination, a process that activates endogenous hydrolytic enzymes, facilitates nutrient mobilization, enhances bioavailability, and alters the nutritional and technological properties of the grain^5,6^. Barley possesses a rich phytochemical composition, encompassing phenolic acids, flavonoids, lignans, tocols, folates, phytosterols, and terpenoids, which contribute to its antioxidant, anti-inflammatory, antidiabetic, anti-arthritic, and cholesterol-lowering activities^2,4^. In addition to these metabolic effects, barley and barley-derived products have been associated with neurofunctional benefits^7^, including modulation of stress-related neurotrophic pathways, attenuation of oxidative stress and pro-inflammatory cytokine levels, and improvement of mitochondrial function^8^. These effects are largely attributed to the polyphenol-rich composition and antioxidant capacity of barley, supporting its relevance as a promising cereal matrix for neuroprotective functional nutrition^9–11^.

Germination is a sustainable bioprocessing strategy that improves the nutritional quality and health-promoting potential of cereal grains^12^. Germination initiates the degradation of storage macromolecules, thereby enhancing the digestibility of starch and proteins. It also increases the bioavailability of essential nutrients, including amino acids, simple sugars, minerals, vitamins, and bioactive compounds such as γ-aminobutyric acid, flavonoids, and phenolic acids, through the activation of endogenous hydrolytic enzymes^13^. These biochemical modifications are associated with an increase in antioxidant capacity, supporting the integration of germinated cereals into functional food products^14,15^. Experimental studies further suggest that sprouted and bioactive-enriched grains may exert neuroprotective effects; pre-germinated brown rice improved spatial learning and attenuated β-amyloid-induced memory deficits in mice^16^, GABA-enriched germinated riceberry improved memory and reduced hippocampal oxidative stress in cerebral ischemia-reperfusion mice^17^, and barley grain improved maze performance in memory-impaired rats^9^. These findings support the premise that germination-derived bioactives contribute to neurological modulation.

Microbial processing has emerged as a valuable biotechnological strategy for improving the nutritional and functional attributes of cereal grains^18^. Conventional approaches, including lactic acid fermentation, solid-state fermentation, and microbial enzymatic treatment, have been widely used to enhance nutrient digestibility, liberate bound phenolics, improve metabolite bioavailability, and strengthen antioxidant potential^19,20^. Within this broader framework, microbial biopriming represents a more targeted seed-level intervention, as it is applied to living seeds during the pre-germination or early germination phases, rather than to processed cereal substrates. During biopriming, beneficial microorganisms are introduced onto the grain surface, where they interact with seeds during active metabolic reprogramming^21^. This early microbe–seed interaction may improve germination performance, stimulate endogenous enzyme activity, promote nutrient mobilization, and enhance the biosynthesis of bioactive metabolites. Mechanistically, biopriming may regulate central metabolic and stress-adaptive pathways, including the tricarboxylic acid cycle^22,23^ and phenylpropanoid biosynthesis, thereby promoting the accumulation of secondary metabolites and other bioactive phytochemicals^24,25^. Accordingly, microbial biopriming provides a specialized platform for enhancing the functional potential of germinated cereals while supporting sustainable crop and food development (SDG2, SDG3, and SDG12) through improved seed vigor, stress resilience, cereal bioactivity, and reduced dependence on chemical inputs^26–29^.

In the present study, *Bacillus subtilis* and *Aspergillus niger* were selected as microbial biopriming agents during barley germination to compare bacterial- and fungal-mediated modulation of cereal metabolism. Previous reports have indicated that *B. subtilis* produces extracellular hydrolytic enzymes that support nutrient mobilization, enhance antioxidant-associated metabolites, and promote seed vigor^30^. In parallel, *A. niger* exhibits a strong capacity to secrete carbohydrases, proteases, cellulases, hemicellulases, and phytases, which contribute to cell wall modification, liberation of bound phenolics, reduction of phytic acid, and improved nutrient availability^31^. Accordingly, the comparative evaluation of *B. subtilis*– and *A. niger*–primed germinated barley provides a relevant model for determining how distinct microbial inoculants differentially reshape the phytochemical composition and functional bioactivity of the barley.

To determine whether these microbially driven phytochemical modifications are associated with biological efficacy, an in vivo model capable of capturing neurobehavioral, neurochemical, and molecular alterations was used. Monosodium glutamate (MSG)-induced neurotoxicity in rats provides a relevant experimental platform for investigating attention-deficit/hyperactivity disorder (ADHD)-like behavioral alterations and associated neurochemical and molecular disturbances^32^. This model recapitulates several pathological features relevant to neurodevelopmental dysfunction, including locomotor hyperactivity, impaired learning and memory, monoaminergic and glutamatergic imbalance, oxidative stress, inflammatory activation, apoptotic injury, autophagic dysregulation, and disruption of neuroprotective signaling pathways^33^. Rat models have also been extensively used to evaluate grain-derived and functional food interventions targeting cognitive performance, cerebral oxidative status, inflammatory mediators, and neurodegeneration-associated biomarkers^9,34^.

In spite of increasing recognition of germinated cereals as functional food matrices, microbial biopriming remains insufficiently investigated as a targeted seed-level approach for improving cereal functionality and neuroprotective potential. Accordingly, the present study evaluated germinated barley bioprimed with *B. subtilis* or *A. niger* to determine how bacterial and fungal treatments differentially modulate the phytochemical profile and biological efficacy of barley. The resulting extracts were examined using complementary chromatographic and spectroscopic analyses, in vitro antioxidant assays, neurobehavioral assessments, neurotransmitter profiling, and molecular investigations in a MSG-induced ADHD-like rat model. This integrated design enabled a direct comparison of the microbial treatments with native germinated barley and allowed the neurobehavioral outcomes to be interpreted in relation to the phytochemical composition, redox homeostasis, inflammatory status, apoptotic regulation, autophagic balance, and associated signaling pathways. By integrating seed-stage microbial intervention with functional and mechanistic validation, this study strengthens the scientific basis for developing germinated barley as a sustainable cereal-derived candidate for neurofunctional-food applications.

## Results

### Phytochemical characterization

The extraction yields differed among the three germinated barley preparations, reaching 10%, 12%, and 9% (*w*/*w*) for native germinated barley extract (GBE), *B. subtilis* bioprimed barley extract (GBB), and *A. niger* bioprimed barley extract (GBF), respectively. GBB showed the highest extraction yield, suggesting that *B. subtilis* biopriming may enhance extract recovery, potentially through enzymatic modification of the seed matrix, improved cell wall disruption, and increased metabolite solubilization during germination. Phytochemical evaluation further demonstrated that microbial biopriming distinctly influenced the compositional profile of the germinated barley. Given the importance of cereal grains as major contributors to global nutritional security and dietary protein intake across diverse food systems^29^, the protein content was first assessed among the tested extracts. Remarkably, GBF exhibited a marked increase in protein content, reaching approximately two-fold higher levels than both native GBE and bacterially bioprimed GBB (49.55%, 20.47%, and 17.33%, respectively). In contrast, the total phenolic content showed a distinct pattern, with GBB displaying the highest concentration (14.78 mg/g), followed by GBE (12.07 mg/g), whereas GBF showed the lowest value (9.22 mg/g). A similar tendency was observed for total flavonoid content, with values of 1.027, 0.941, and 0.633 mg/g in GBE, GBB, and GBF, respectively. These findings underscore the differential modulation of phytochemical composition by distinct microbial treatments: bacterial biopriming selectively augmented phenolic biosynthesis, whereas fungal biopriming substantially amplified protein levels at the apparent expense of phenolic and flavonoid content, highlighting the potential for targeted microbial intervention to tailor the nutritional and bioactive profiles of germinated barley (Fig. S1 and Supplementary File).

**Fig. 1:**
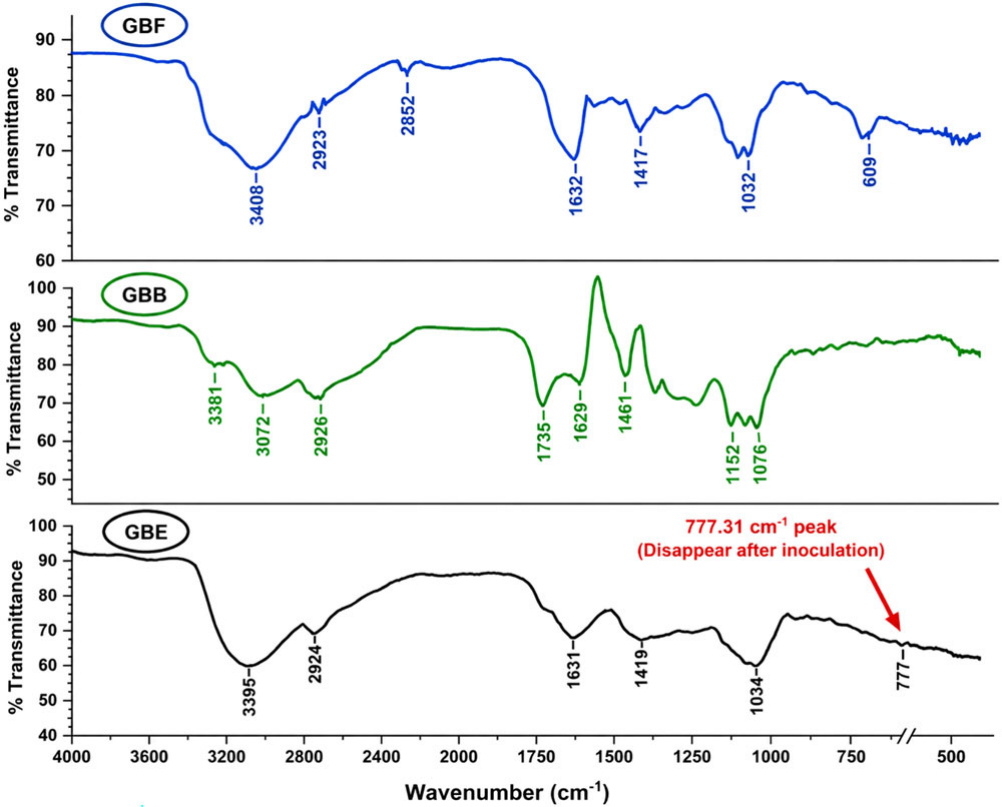
FTIR spectral characterization of germinated barley extracts. FTIR spectral profi les of GBE, GBB, and GBF, highlighting characteristic functional group variations and chemical fi ngerprints among the three extracts.

Fourier-transform infrared (FTIR) spectroscopic analysis of the GBEs (GBE, GBB, and GBF) revealed distinct functional group variations, reflecting the microbial strain-specific biochemical remodeling of the native sample, GBE (Fig. 1). A pronounced divergence was observed within the fingerprint region (1500–500 cm⁻¹), a diagnostically sensitive zone that reflects structural changes in carbohydrates, phenolic compounds, and proteins^35–37^. The untreated extract (GBE) exhibited a sharp band at 777 cm⁻¹, characteristic of aromatic or heterocyclic ring vibrations, which disappeared following microbial inoculation (GBB and GBF), suggesting microbial transformation or degradation of the native aromatic structures. The *B. subtilis*-primed extract (GBB) displayed a cluster of complex peaks between 1000 and 1200 cm⁻¹, attributed to the C–O stretching vibrations of glycosidic linkages. This pattern implies enhanced carbohydrate metabolism and microbial remodeling of polysaccharide chains, involving cleavage, rearrangement, or transglycosylation reactions, facilitated by bacterial glycosidases and transglycosylases^38–40^. Conversely, the *A. niger* bioprimed extract (GBF) exhibited a distinct band at 669 cm⁻¹, associated with aromatic out-of-plane bending, indicating fungal-mediated oxidative polymerization and structural diversification^35^. Across all extract spectra, absorption bands at 2920–2850 cm⁻¹ corresponded to aliphatic –CH₂ and –CH₃ stretching vibrations, confirming the presence of a shared hydrocarbon backbone^41^. A broad band near 1000 cm⁻¹ reflects the overlapping C–O, C–C, and C–OH stretching and bending vibrations^42^. The O–H stretching region (3268–3361 cm⁻¹) showed variable intensities and red shifts, consistent with the differences in hydrogen bonding environments. Specifically, the relatively higher O–H peak intensity in GBE (3350 cm⁻¹) suggests a less constrained molecular network, whereas the red shift in GBB (3304 cm⁻¹) and GBF (3296 cm⁻¹) indicates stronger intermolecular hydrogen bonding^43^. The amide I band shifted from 1604 cm⁻¹ (GBE) and 1606 cm⁻¹ (GBB) to 1629 cm⁻¹ in GBF, which may reflect alterations in the protein secondary structure, such as increased β-sheet content^44^. Additionally, GBB exhibited a strong absorption at 1729 cm⁻¹, corresponding to the C = O stretching of carbonyl groups (esters, aldehydes, or carboxylic acids), reflecting *Bacillus*-mediated oxidation and esterification processes. In contrast, the moderate bands at 1600–1630 cm⁻¹ in GBE and GBF were assigned to aromatic C = C and amide I vibrations, indicating protein–phenolic conjugation^45^. Hence, FTIR spectral analysis indicated that microbial biopriming induced strain-dependent functional group remodeling in the GBEs. Although the three samples shared core spectral features, *B. subtilis* biopriming (GBB) suggested the relative enrichment of carbonyl-bearing moieties, whereas non-bioprimed GBE retained a more pronounced aromatic profile. In contrast, *the A. niger* bioprimed extract (GBF) appeared to promote greater chemical heterogeneity, with enhanced intermolecular interactions and possible conformational reorganization. These changes were reflected by shifts in the FTIR bands associated with hydroxyl, carbonyl, amide, and polysaccharide vibrations, suggesting modifications in hydrogen bonding, protein-associated structures, and carbohydrate-related functional groups. However, these data should be interpreted as supportive spectroscopic evidence rather than definitive structural definitions.

High-performance liquid chromatography (HPLC) analysis enabled the identification of 16 phenolic constituents across GBE, GBB, and GBF, with GBB displaying the most diverse and quantitatively enriched phenolic profile relative to the other extracts (Table 1, Fig. S2, and Supplementary File). Remarkably, syringic acid (1744 ± 12 µg/g) and rutin (1618 ± 38 µg/g) were markedly elevated in GBB, representing approximately 45- and 24-fold increases, respectively, compared to GBE. GBB was also enriched in ellagic acid (237 ± 7.6 µg/g), methyl gallate (29.5 ± 3.1 µg/g), and caffeic acid (500 ± 6.7 µg/g), indicating a pronounced enhancement of hydroxybenzoic and hydroxycinnamic acid derivatives. In contrast, GBF exhibited a more moderate phenolic profile, with generally lower concentrations of most detected compounds and the absence of several constituents, including catechin, rosmarinic acid, daidzein, and hesperidin. However, GBF showed comparable or slightly higher levels of selected bioactives, particularly naringenin (38 ± 2.1 µg/g) and quercetin (50 ± 3.4 µg/g), compared to GBE. The non-bioprimed GBE displayed a baseline phenolic profile, with catechin (134 ± 6.2 µg/g) and caffeic acid (265 ± 5.5 µg/g) as predominant constituents.

**Table 1:** Chromatographic profiling of phenolic compounds in aqueous extracts of germinated barley and its bio-primed derivatives (GBE, GBB, and GBF), monitored at 180–480 nm (λmax)

Polyphenol	Calibration curves	LOQ^*^ (µg/ml)	LOD^**^ (µg/ml)	Concentration(µg/g)^*^		
Analytes				GBE	GBB	GBF
Gallic acid	*y* = 13.897*x* − 4.2378	1.25	0.41	3548 ± 47	3485 ± 13	2891 ± 4.9 ^ab^
Chlorogenic acid	*y* = 7.1361*x* + 2.8237	3.12	1.04	1344 ± 29	950 ± 9^a^	1025 ± 15 ^ab^
Catechin	*y* = 4.6927*x* + 1.3675	4.68	1.42	134 ± 6.2	102 ± 8.7^a^	ND^a, b^
Methyl gallate	*y* = 17.678*x* + 2.172	0.93	0.28	8.6 ± 0.84	29.5 ± 3.1^a^	15 ± 1.4^b^
Caffeic acid	*y* = 19.307*x* + 2.8803	1.25	0.37	265 ± 5.5	500 ± 6.7^a^	234 ± 5.5^a, b^
Syringic acid	*y* = 16.846*x* + 3.1328	1.25	0.37	39 ± 4.6	1744 ± 12^a^	29 ± 5.3^b^
Rutin	*y* = 6.4554*x* + 0.7902	3.12	1.04	68 ± 4.3	1618 ± 38^a^	130 ± 11^b^
Ellagic acid	*y* = 10.019*x* + 0.9525	4.37	1.32	19 ± 2	237 ± 7.6^a^	28 ± 5.3^b^
Coumaric acid	*y* = 27.51*x* + 4.1743	1.25	0.41	52 ± 4.3	18 ± 4^a^	16 ± 4.2^a^
Vanillin	*y* = 27.246*x* + 4.4144	1.25	0.37	37 ± 2.9	71 ± 5.1^a^	27 ± 3.1^b^
Ferulic acid	*y* = 17.057*x* + 2.7067	1.25	0.38	51 ± 4.1	120 ± 4.9^a^	21 ± 3.5^a, b^
Naringenin	*y* = 10.728*x* + 2.726	1.87	0.56	34 ± 5.9	25 ± 1.5	38 ± 2.1
Rosmarinic acid	*y* = 10.192*x* + 3.9557	3.12	0.94	41 ± 2.1	52 ± 1.2^a^	ND^a, b^
Daidzein	*y* = 17.335*x* + 1.7923	1.25	0.37	ND	25 ± 1.8^a^	ND^b^
Quercetin	*y* = 7.905*x* + 1.13898	2.51	0.76	32 ± 3.5	64 ± 4.7^a^	50 ± 3.4^a^
Cinnamic acid	*y* = 50.836*x* + 4.6567	0.62	0.20	30 ± 3.6	8.6 ± 2^a^	3.4 ± 0.92^a^
Kaempferol	*y* = 39.433*x* + 6.8241	1.26	0.38	61 ± 3.3	45 ± 2.9^a^	25 ± 2.9^a, b^
Hesperidin	*y* = 21.272*x* + 4.3652	1.25	0.37	ND	19 ± 3.5 ^s^	ND^b^

*ND* not detected, *GBB* *B. subtilis* bioprimed barley extract, *GBE* germinated barley extract, *GBF* *A. niger* bioprimed barley extract, LOD** limit of detection, LOQ* limit of quantification

^a,b^Values considered significant when compared to the GBE and GBB groups, respectively. One-way ANOVA followed by Tukey’s as a post-hoc test presented *P*-value < 0.05, *R*^2^ = 0.999

^*^Results are presented as mean ± SEM of three determinations.

Amino acid profiling revealed significant compositional differences among the three GBEs (GBE, GBB, and GBF), as shown in Table 2, Fig. S3, and Supplementary File. Compared with GBE and GBB, GBF exhibited markedly higher levels (*P* < 0.05) of glutamate (74.0 ± 2.4 mg/g), aspartate (12.4 ± 1.2 mg/g), serine (9.2 ± 0.6 mg/g), and threonine (10.4 ± 0.5 mg/g), all of which are closely involved in nitrogen assimilation, amino acid interconversion, and protein biosynthesis. Several amino acids were either uniquely detected or substantially enriched in the GBF. Notably, arginine (8.6 ± 0.8 mg/g) and histidine (6.9 ± 0.2 mg/g), which were not detected in GBE or GBB, were present at considerable levels in GBF, suggesting the activation of distinct biosynthetic or proteolytic pathways associated with fungal enzymatic metabolism. GBF also showed increased concentrations of essential amino acids, including isoleucine (11.8 ± 0.3 mg/g), methionine (6.6 ± 0.2 mg/g), lysine (9.5 ± 0.4 mg/g), and valine (8.0 ± 0.1 mg/g), indicating an improved amino acid profile and enhanced nutritional value of the product. The *B. subtilis* bioprimed extract (GBB) showed moderate increases in selected amino acids relative to GBE, including alanine (12.2 ± 0.6 mg/g), glycine (3.3 ± 0.1 mg/g), and valine (6.0 ± 0.1 mg/g). In contrast, GBE displayed the lowest amino acid content across most detected compounds, with arginine, histidine, and cysteine being undetectable. These findings indicate that *A. niger* biopriming was more effective than bacterial biopriming in enhancing the amino acid composition of germinated barley, likely through fungal proteolytic enzyme activity and modulation of nitrogen metabolism^46^.

**Table 2:** Chromatographic profiling of aqueous extracts of amino acids from germinated barley (GBE) and its microbially bio-primed derivatives: GBB and GBF

Amino acids			Concentrations (mg/g)		
	Calibration curves	*R*^2^	GBE	GBB	GBF
Aspartate	*y* = 0.0723*x* + 1.1861	0.9976	4.3 ± 0.2	5.4 ± 0.4	12.4 ± 1.2^a,b^
Glutamate	*y* = 0.0670*x* + 0.2867	0.9999	30.4 ± 1.4	42.3 ± 1.5^a^	74 ± 2.4^a,b^
Serine	*y* = 0.0395x + 0.973	0.9998	2.3 ± 0.1	2.6 ± 0.2	9.2 ± 0.6^a,b^
Histidine	*y* = 0.014*x* − 17.095	0.9948	ND	2.9 ± 0.2^a^	6.9 ± 0.2^a,b^
Glycine	*y* = 0.0549*x* − 3.331	0.9994	2.0 ± 0.1	3.3 ± 0.1^a^	7.6 ± 0.4^a,b^
Threonine	*y* = 0.0376*x* + 4.685	1	3.7 ± 0.3	4.8 ± 0.1	10.4 ± 0.5^a,b^
Arginine	*y* = 0.029*x* − 4.9812	0.9999	ND	ND	8.6 ± 0.8^a,b^
Alanine	*y* = 0.0506*x* + 0.081	0.9998	5.3 ± 0.3	12.2 ± 0.6^a^	13.3 ± 0.6^a^
Tyrosine	*y* = 0.0245*x* − 6.006	0.9999	5.4 ± 0.3	6.6 ± 0.4	8.5 ± 0.2^a,b^
Cystine	*y* = 0.0008*x* − 0.249	0.9975	ND	ND	ND
Valine	*y* = 0.0535*x* + 0.977	1	4.7 ± 0.2	6 ± 0.1^a^	8.0 ± 0.1^a,b^
Methionine	*y* = 0.0365*x* + 0.411	0.9999	3.6 ± 0.2	3.8 ± 0.2	6.6 ± 0.2^a,b^
Phenylalanine	*y* = 0.0291*x* − 0.717	0.9997	6.5 ± 0.2	6.5 ± 0.2	8.9 ± 0.1^a,b^
Isoleucine	*y* = 0.0415*x* − 2.302	0.9999	8.4 ± 0.3	8.7 ± 0.3	11.8 ± 0.3^a,b^
Leucine	y = 0.0396*x* − 6.595	0.9998	11.3 ± 0.2	11.4 ± 0.3	12.9 ± 0.8
Lysine	*y* = 0.0026*x* + 8.154	0.9996	7 ± 0.2	6.5 ± 0.3	9.5 ± 0.4^a,b^
Proline	*y* = 0.0221*x* + 14.77	0.9976	12.4 ± 0.3	9.4 ± 0.3^a^	14.5 ± 0.4^a,b^

The limit of quantification (LOQ) for amino acid determination was 10 nmol/ml.

*GBB* *B. subtilis* bioprimed barley extract, *GBE* germinated barley extract, *GBF* *A. niger* bioprimed barley extract, *ND* not detected.

^a,b^Values considered significant when compared to the GBE and GBB groups, respectively. One-way ANOVA followed by Tukey’s as a post-hoc test presented *P*-value < 0.05.

^*^Results are presented as mean ± SEM of three determinations.

HPLC analysis revealed pronounced treatment-dependent differences in the comparative B-vitamin profiles of the GBEs (Table 3 and Fig. S4). GBE exhibited a baseline vitamin profile dominated by thiamine (B1; 1402 ± 2.6 µg/g), whereas riboflavin (B2), niacin (B3), folate (B9), and cobalamin (B12) were detected at comparatively lower concentrations. Microbial biopriming differentially alters the B-vitamin profile in a strain-specific manner. Under the applied analytical conditions, GBB exhibited the greatest relative abundance of vitamins B2, B9, and B12, with approximately 53-fold, 3.9-fold, and 13-fold higher responses, respectively, compared to GBE. In contrast, GBF showed relatively higher levels of thiamine (B1) and pyridoxine (B6), with B1 increasing by approximately 1.5-fold, and B6 was detected only in the fungal-bioprimed extract. These findings indicate that microbial biopriming differentially modulates the comparative B-vitamin profile of germinated barley, with *B. subtilis* preferentially associated with the relative enrichment of riboflavin, folate, and cobalamin, whereas *A. niger* favors thiamine- and pyridoxine-associated vitamin profiles. Because B-vitamin quantification was performed using single-point external calibration, these findings should be interpreted as comparative differences among treatments rather than definitive absolute vitamin concentrations.

**Table 3:** Concentrations of B vitamins in the GBE, GBB and GBF extracts by HPLC analysis at λ 280 nm

Vitamins	Concentrations (*µ*g/g)		
	GBE	GBB	GBF
B1	1402 ± 2.6	734.2 ± 6.2^a^	2092 ± 4.6^a,b^
B2	17 ± 1.4	901 ± 3.4^a^	112 ± 4.3^a,b^
B3	33 ± 3.7	ND^a^	13±2^a,b^
B6	ND	ND	1384± 6^a,b^
B9	28 ± 1.5	109 ± 2^a^	111 ± 2.4^a^
B12	58 ± 5	756 ± 17^a^	155.3 ± 6.3^a,b^

Quantification was performed using a single-point calibration.

*GBE* germinated barley extract, *GBB* *B. subtilis* bioprimed barley extract, *GBF* *A. niger* bioprimed barley extract, *ND* not detected.

^a,b^Values considered significant when compared to the GBE and GBB groups, respectively. One-way ANOVA followed by Tukey’s as a post-hoc test presented *P*-value < 0.05.

^*^Results are presented as mean ± SEM of three determinations.

**Fig. 2:**
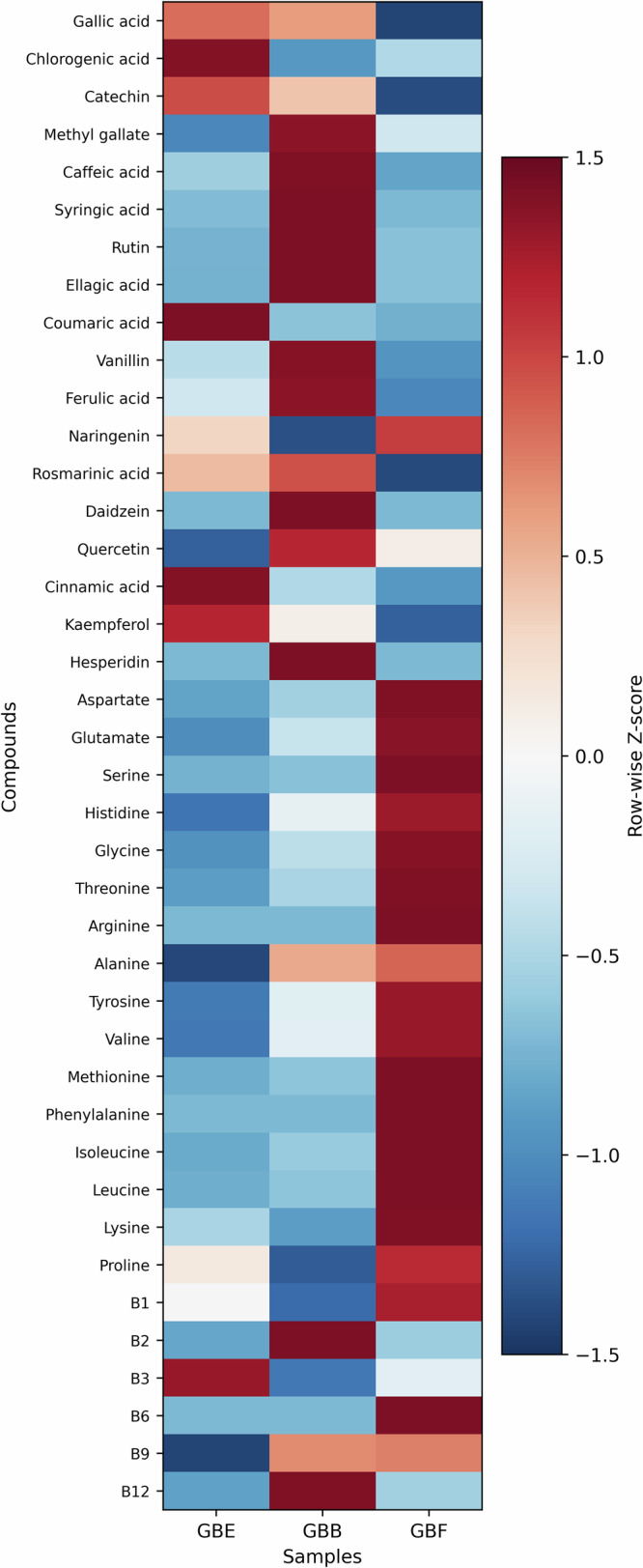
Comparative visualization of metabolite abundance patterns in GBEs following microbial biopriming. Heatmap showing the row-wise *Z*-score-normalized abundance of amino acids, B vitamins, and phenolic compounds in untreated GBE, GBB, and GBF. Each metabolite was normalized independently across the three treatments to facilitate comparison of relative abundance patterns irrespective of differences in concentration ranges and measurement units. Color intensity represents the relative abundance of each metabolite, highlighting the distinct, strain-dependent metabolic remodeling induced by microbial biopriming.

Heatmap visualization of row-wise *Z*-score-normalized metabolite abundances revealed clear treatment-dependent metabolic differentiation among the three GBEs (Fig. 2). GBB displayed a distinct metabolic signature characterized by the relative enrichment of several phenolic compounds, particularly syringic acid, rutin, caffeic acid, and ellagic acid, along with vitamins B2 and B12, indicating the preferential enhancement of phenolic metabolism and selected B-vitamin biosynthetic pathways following bacterial biopriming. In contrast, GBF exhibited a metabolic profile dominated by the relative enrichment of free amino acids, including glutamate, aspartate, serine, threonine, arginine, histidine, lysine, and proline, which is consistent with enhanced protein turnover, nitrogen mobilization, and fungal enzyme-mediated metabolic remodeling during germination. GBF also showed preferential enrichment of vitamins B1 and B6, suggesting enhanced thiamine- and pyridoxine-associated metabolic pathways, respectively. The non-bioprimed GBE exhibited a comparatively balanced metabolite profile, representing the baseline metabolic state associated with germination. These findings demonstrate that microbial biopriming drives strain-dependent metabolic reprogramming in germinated barley, resulting in distinct phytochemical signatures characterized by preferential phenolic and selected B-vitamin enrichment following *B. subtilis* treatment and enhanced amino acid accumulation following *A. niger* treatment.

### In vitro antioxidant activity

The in vitro antioxidant capacity of the GBE and its bioprimed derivatives was evaluated using the DPPH radical-scavenging assay, with ascorbic acid as the reference standard. The half-maximal inhibitory concentration (IC₅₀) values revealed that all extracts possessed substantial free radical scavenging activity, although with differing potencies. The GBF extract exhibited the strongest antioxidant effect, with the lowest IC₅₀ value (20.37 ± 0.6 mg/ml), followed by GBE (27.00 ± 2.1 mg/ml). GBB demonstrated comparatively lower activity (51.40 ± 1.8 mg/ml) than GBE and GBF. Remarkably, all three extracts outperformed the ascorbic acid control (62 ± 2.5 mg/ml), underscoring the potent antioxidant potential of germinated and microbially transformed barley extracts.

### Behavioral and cognitive restoration by bioprimed barley extracts

**Fig. 3:**
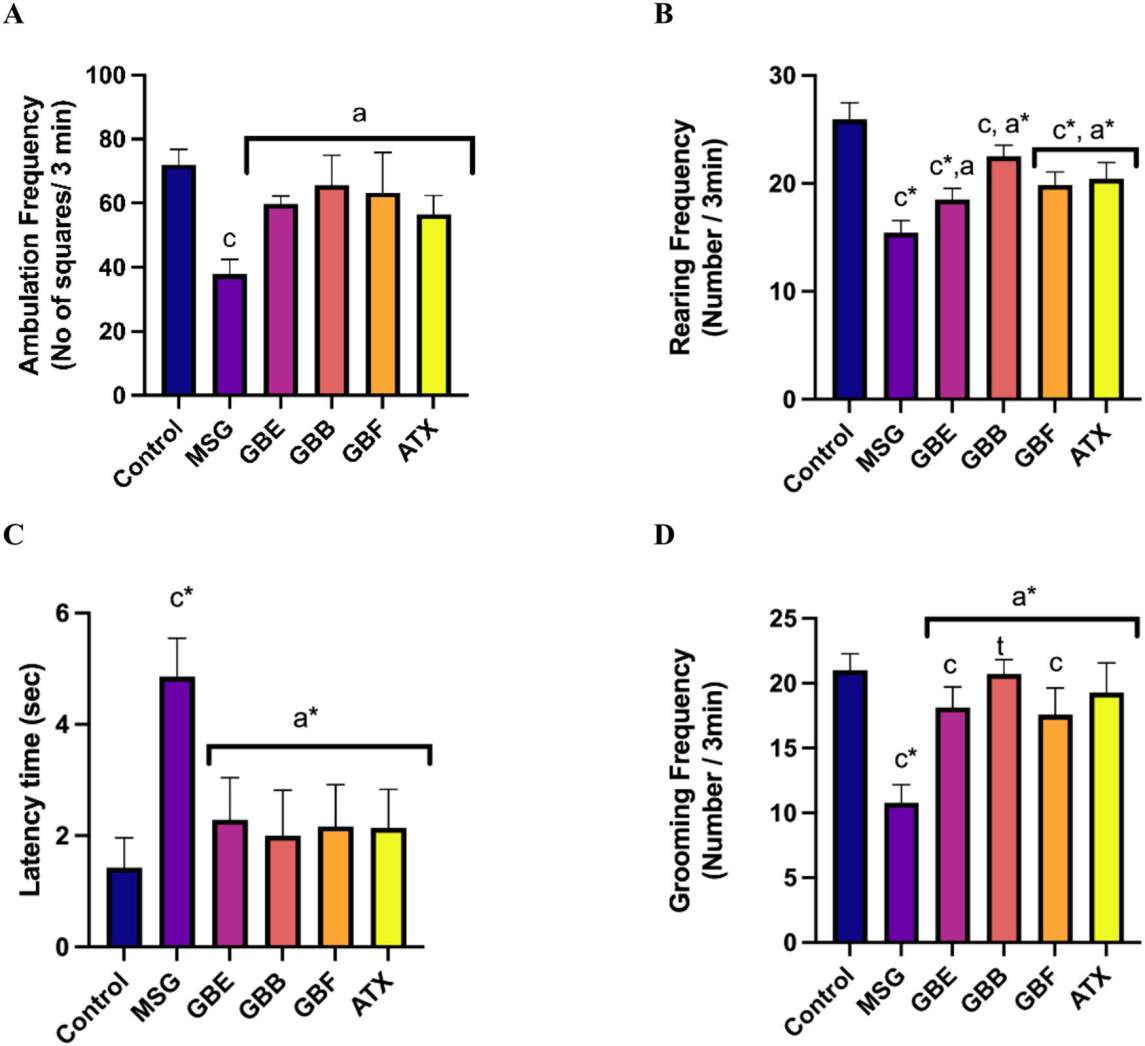
Effects of GBE, GBB, GBF, and ATX on behavioral performance in the open-field test in MSG-treated rats. **A** Ambulation frequency, **B** rearing frequency, **C** latency time, and **D** grooming frequency. Data are presented as the mean ± SD (*n* = 10). Statistical significance was determined using one-way ANOVA followed by Tukey’s post hoc test. ^c^Indicates a significant difference compared with the Control group; ^a^Indicates a significant difference compared with the MSG group; and ^t^Indicates a significant difference compared with the ATX group (*P* < 0.05). ^*^Denotes high statistical significance (*P* < 0.001) for the corresponding comparison.

**Fig. 4:**
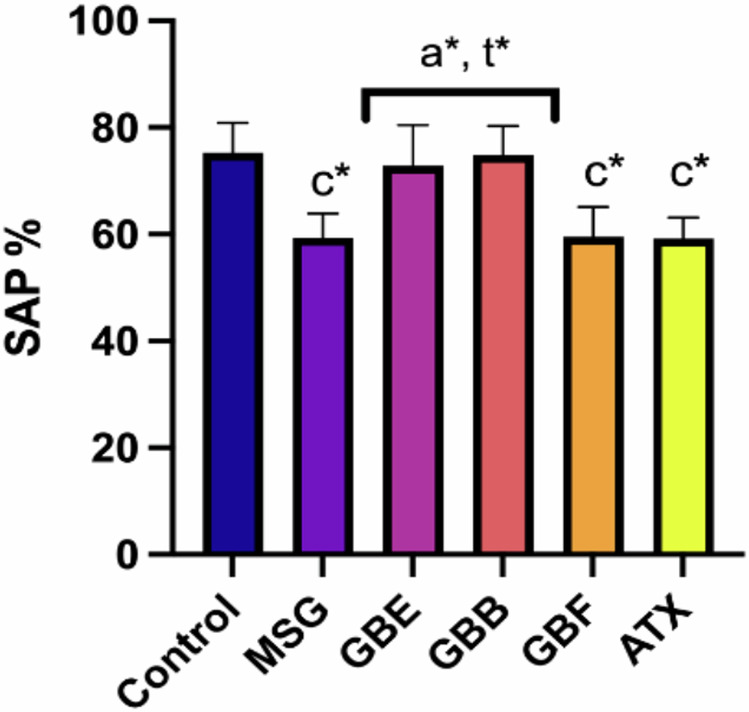
Effect of GBE, GBB, and GBF on SAP (%) in the Y-Maze test as an indicator of learning response in MSG-treated rats. Data are represented as mean ± SD (*n* = 10). ^c^Indicates a significant difference compared with the Control group; ^a^indicates a significant difference compared with the MSG group; and ^t^indicates a significant difference compared with the ATX group. Significance: *P* < 0.05, highly significant, ^*^P< 0.001.

The effects of GBE, GBB, and GBF on MSG-induced ADHD-like behavioral alterations were evaluated by assessing locomotor activity, exploratory behavior, and cognitive performance. In the open-field test, treatment with GBE, GBB, and GBF significantly improved behavioral performance relative to the MSG group, as evidenced by increased ambulation, rearing, and grooming frequencies, together with reduced latency time (Fig. 3). In addition, the spontaneous alternation percentage (SAP%) was significantly reduced by 25% in the MSG group compared with that in the control group, indicating impaired spatial working memory and learning behavior. This deficit was markedly attenuated following treatment with GBE and GBB. In contrast, neither GBF nor atomoxetine (ATX) produced a comparable improvement in spatial working memory compared to the MSG-treated group (Fig. 4).

### Brain neurotransmitter indices

**Fig. 5:**
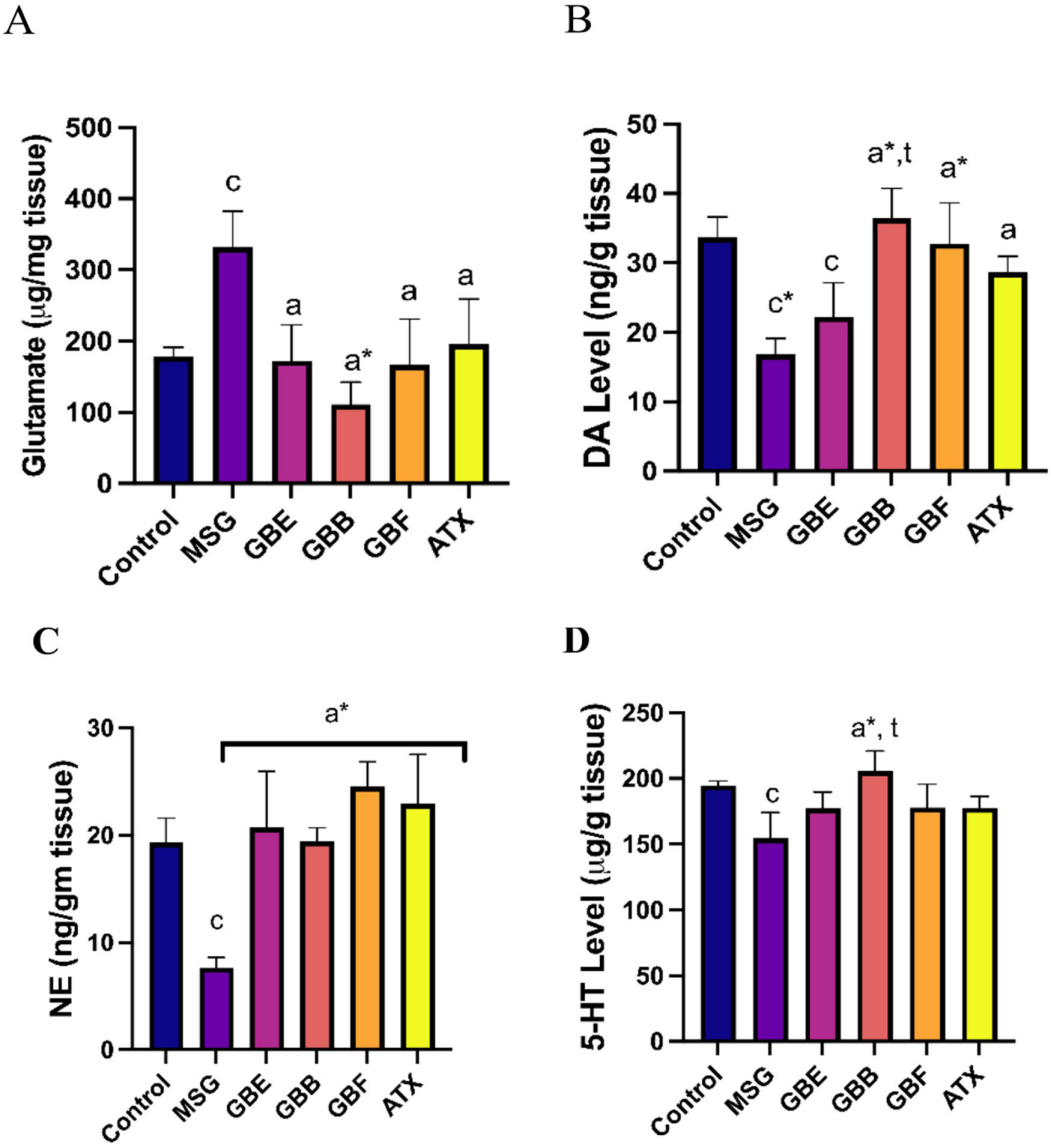
Illustrates that 5: impact of GBE, GBB, and GBF on MSG-induced alterations in brain neurotransmitter Levels. **A** Glutamate level (μg/mg tissue), **B** DA level (ng/g tissue), **C** NE (ng/g tissue), and **D** 5-HT level (μg/g tissue). Data are represented as mean ± SD (*n* = 6). ^c,a,^^t^Indicate significant differences compared with the control, MSG, and ATX groups, respectively (*P* < 0.05). ^*^P < 0.001.

MSG exposure profoundly disrupted cerebral neurotransmitter homeostasis compared to the control group, as evidenced by a 1.85-fold elevation in glutamate levels accompanied by substantial reductions in dopamine (DA), norepinephrine (NE), and serotonin (5-HT) levels by 50%, 60%, and 20%, respectively. Treatment with GBE, GBB, and GBF mitigated these neurochemical perturbations, with GBB showing the most pronounced restorative effect. Compared with the MSG-treated group, GBB reduced glutamate levels by 66.5% and increased DA, NE, and 5-HT levels by 2.17-, 2.50-, and 1.30-fold, respectively, indicating a marked re-establishment of excitatory and monoaminergic neurotransmitter balance (Fig. 5).

### Dysregulation of, α-synuclein levels, and antioxidant status

**Fig. 6:**
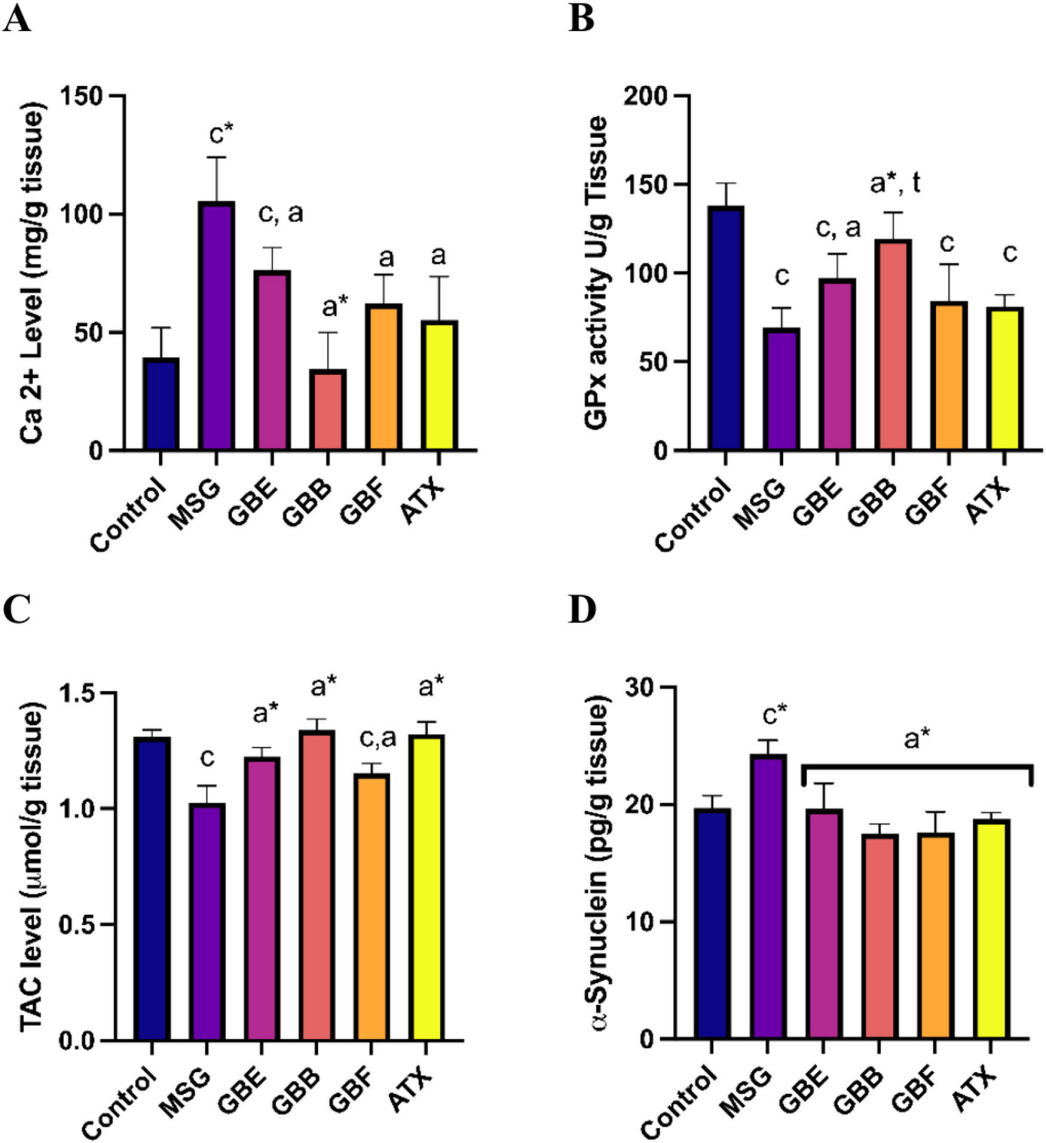
Effects of GBE, GBB, GBF, and ATX on oxidative stress and neurodegeneration biomarkers in MSG-treated rats. **A** Ca²⁺ level (mg/g tissue), (**B**) glutathione peroxidase (GPx) activity (U/g tissue), (**C**) total antioxidant capacity (TAC; μmol/g tissue), and (**D**) α-synuclein level (pg/g tissue). Data are presented as the mean ± SD (*n* = 7). Statistical significance was determined using one-way ANOVA followed by Tukey’s post hoc test. c, a, and t indicate significant differences compared with the Control, MSG, and ATX groups, respectively (*P* < 0.05) *P < 0.001.

MSG exposure induced a marked elevation in cerebral Ca²⁺ levels relative to the control group, accompanied by substantial attenuation of antioxidant defense mechanisms, as reflected by significant reductions in GPx activity by 50% (*F*(5,30) = 20.86, *P* < 0.0001) and TAC by 23% (*F*(5,30) = 39.13, *P* < 0.0001). Concurrently, α-synuclein levels were significantly increased by 23.4% (*F*(5,26) = 22.51, *P* < 0.0001), indicating MSG-induced dysregulation of calcium homeostasis, redox equilibrium, and protein aggregation stress. Treatment with GBE, GBB, and GBF mitigated these alterations, with GBB having the most pronounced protective effect. Notably, GBB demonstrated superior restoration of Ca²⁺ balance and antioxidant capacity compared with ATX, indicating a stronger capacity to preserve brain tissue homeostasis under MSG-induced neurotoxic conditions (Fig. 6).

### Histological evidence of neuroprotection

**Fig. 7:**
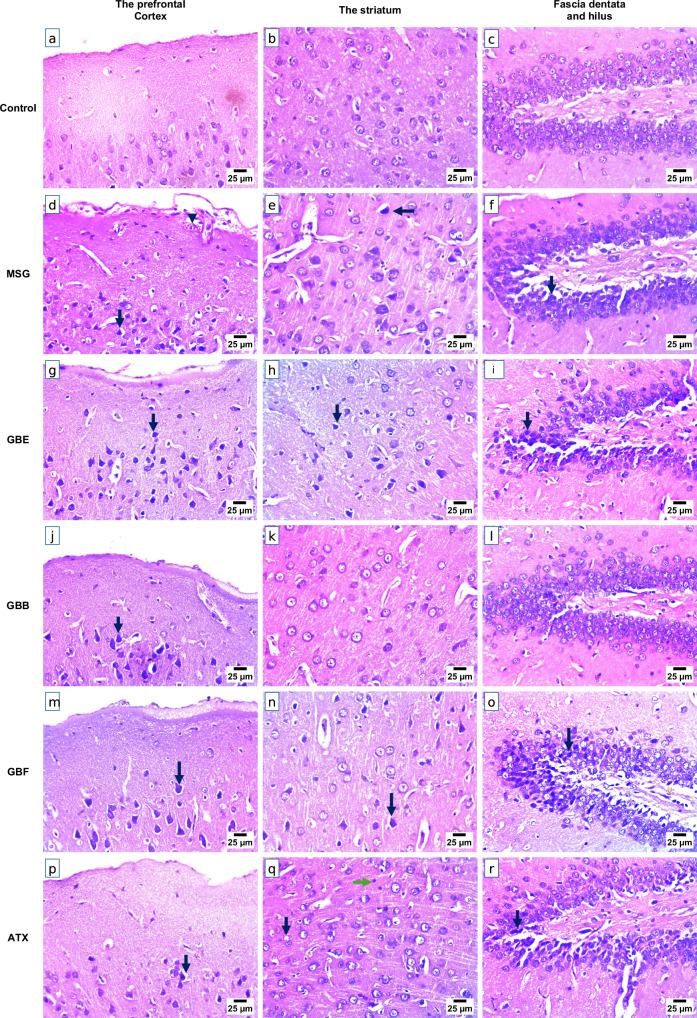
The effect of concurrent treatment with GBE, GBB, GBF, or ATX on MSG-induced histopathological alterations in the brain. Representative hematoxylin and eosin (H&E)-stained brain sections (50× magnification) showing the prefrontal cortex (**a, d, g, j, m, p**), the striatum (**b, e, h, k, n, q**), and the fascia dentata and hilus of the hippocampus (**c, f, i, l, o, r**) from the Control (**a-c**), MSG (**d-f**), GBE (**g-i**), GBB (**j-l**), GBF (**m-o**), and ATX (**p-r**) groups, respectively, illustrating the histopathological changes in each experimental group.

Histopathological examination of brain tissues from MSG-treated rats revealed meningeal hemorrhage, neuronal shrinkage with nuclear pyknosis, and perineuronal edema in the prefrontal cortex and striatum, as well as in neurons of the fascia dentata hilus (Fig. 7d–f). Treatment with GBE or GBF failed to reverse these pathological alterations (Fig. 7g–i, m–o). In contrast, GBB treatment restored the normal histological architecture in the striatum, fascia dentata, and hilus, with only residual nuclear pyknosis persisting in the prefrontal cortex (Fig. 7j–l). ATX treatment resulted in mild histological improvement across the examined regions (Fig. 7p–r). The degree of structural recovery observed with GBB surpassed that of GBE, GBF, and ATX, consistent with the enhanced neuroprotective capacity conferred by *B. subtilis*-mediated biopriming.

### Mechanistic pathway of the most bioactive bioprimed extract, GBB

**Fig. 8:**
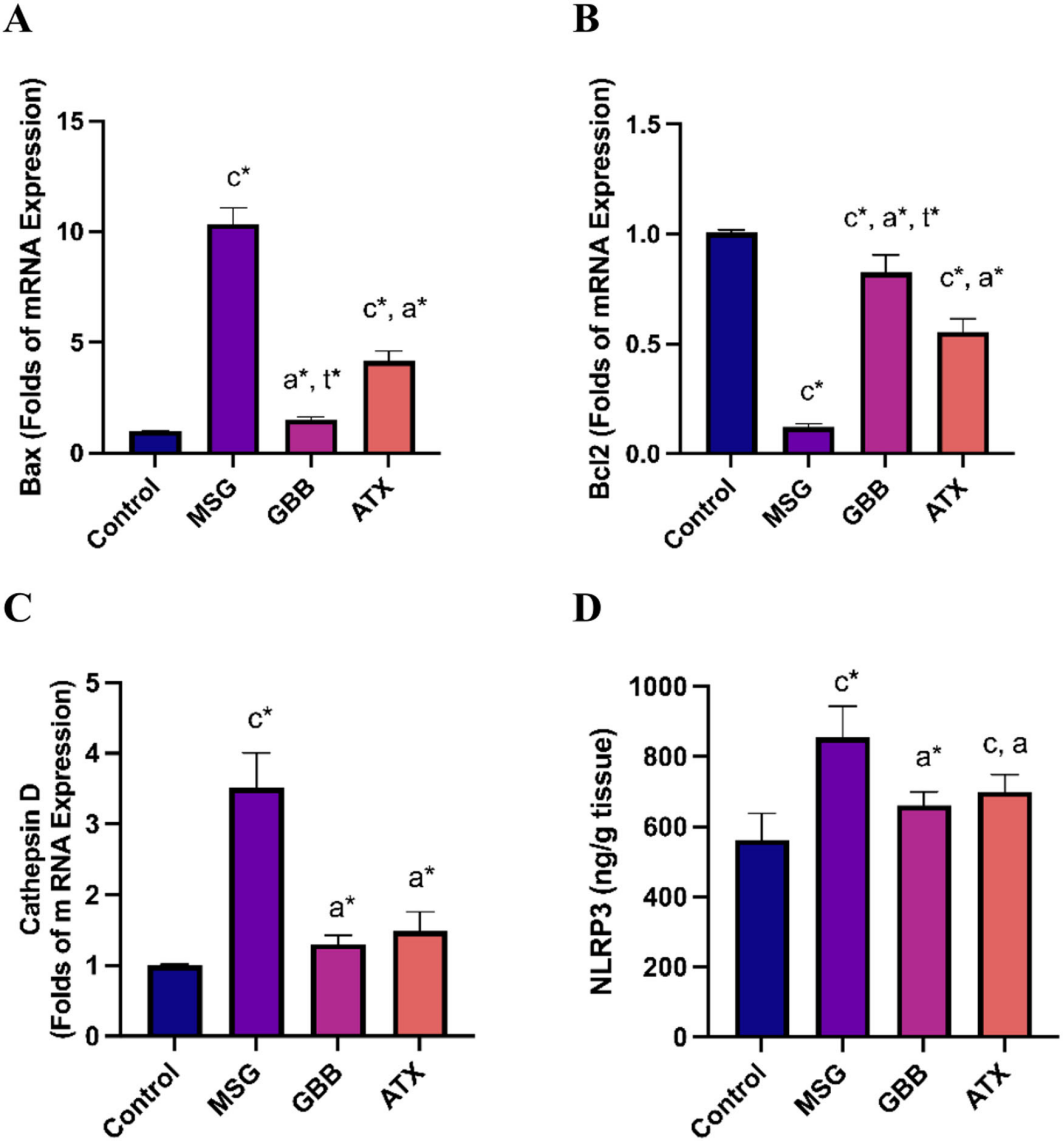
Effect of GBB treatment on apoptosis- and inflammasome-related gene expression in MSG-treated rats. Relative gene expression of **A** BAX, **B** Bcl-2, **C** Cathepsin D, and **D** NLRP3. Data are presented as the mean ± SD (*n* = 6). Statistical significance was determined using one-way ANOVA followed by Tukey’s post hoc test. c, a, and *t* indicate significant differences compared with the Control, MSG, and ATX groups, respectively (*P* < 0.05) *P < 0.001.

**Fig. 9:**
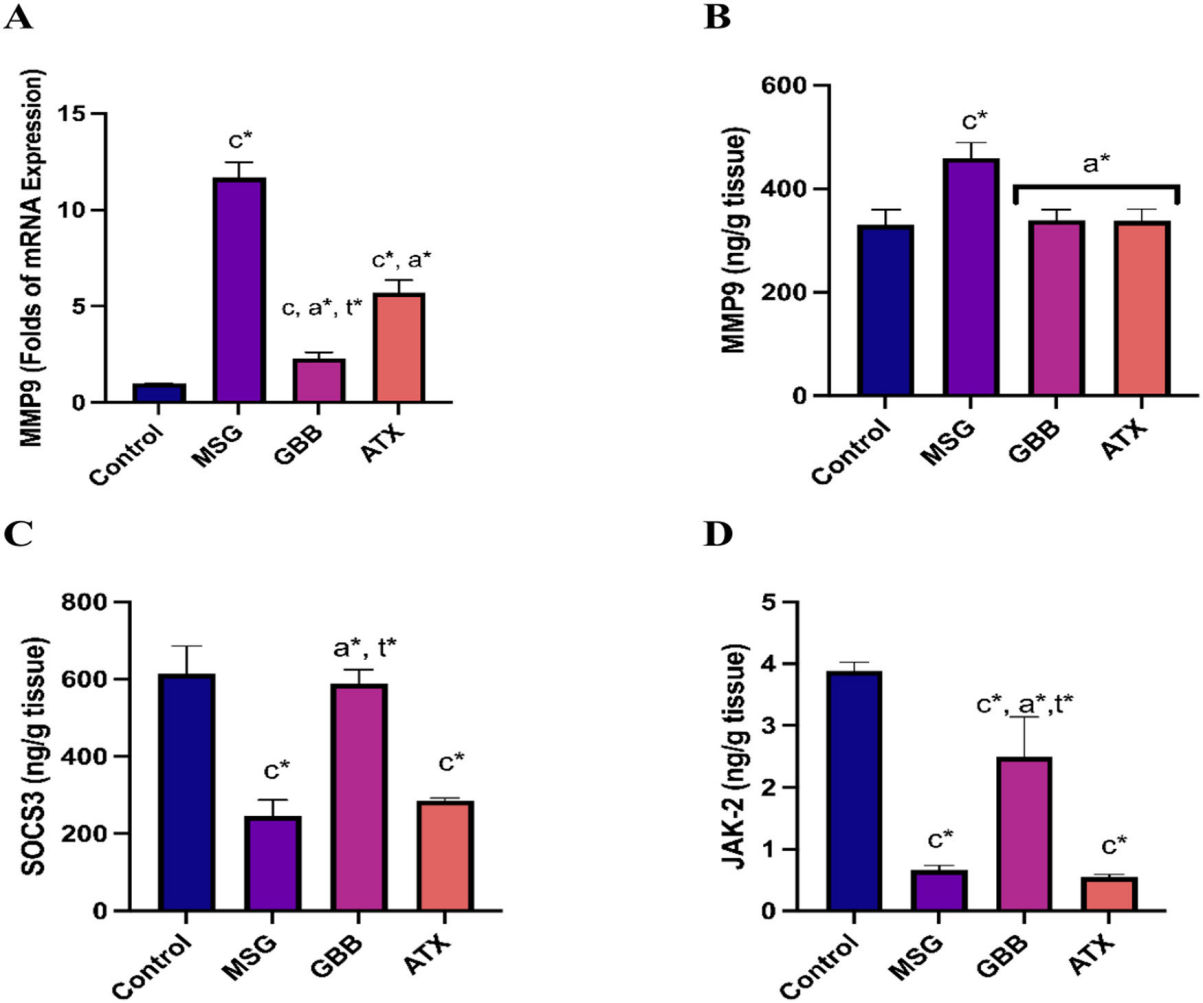
The effect of GBB treatment on the inflammatory action induced by MSG. **A** MMP9 (Folds of mRNA expression), **B** MMP9 (ng/g tissue), **C** SOCS3 (ng/g tissue), and **D** JAK-2 (ng/g tissue). Data are represented as mean ± SD (*n* = 6). ^c,a,^^t^Indicate significant differences compared with the Control, MSG, and ATX groups, respectively (*P* < 0.05). ^*^P < 0.001.

**Fig. 10:**
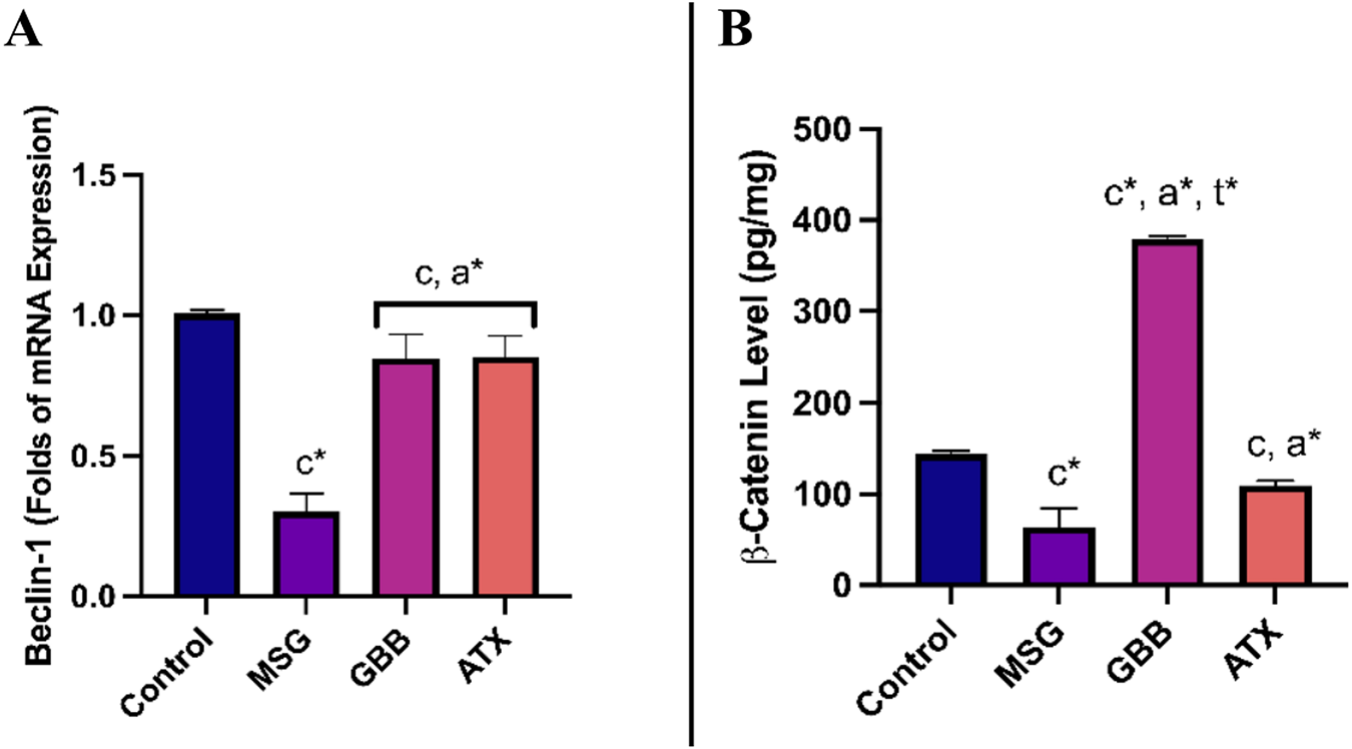
Effect of GBB on Beclin-1 and β-Catenin in MSG-treated rats. **A** Beclin-1 (folds of mRNA expression) and **B** β-Catenin level (pg/mg tissue). Data are represented as mean ± SD (*n* = 6). ^c^Significantly different from the control group. ^c,a,^^t^Indicate significant differences compared with the Control, MSG, and ATX groups, respectively (*P* < 0.05). ^*^P < 0.001.

Given GBB’s comparatively superior neuroprotective efficacy and more pronounced histopathological improvement relative to GBE and GBF, it was selected for subsequent mechanistic investigations. MSG exposure markedly promoted apoptotic cell death, as evidenced by a substantial increase in BAX mRNA expression by 1000% (*F*(3,21) = 587.5, *P* < 0.0001) and a concomitant reduction in Bcl-2 mRNA expression by 88% (*F*(3,21) = 392.4, *P* < 0.0001) compared to the control group. MSG administration also significantly upregulated cathepsin D mRNA expression by 350% (*F*(3,21) = 95.73, *P* < 0.0001) and increased NLRP3 levels by 152% (*F*(3,20) = 20.04, *P* < 0.0001), indicating the activation of apoptotic, lysosomal, and inflammasome pathways. Treatment with GBB effectively reversed these MSG-induced molecular alterations, accompanied by significant reductions in cathepsin D expression and NLRP3 levels by 63% and 23%, respectively, compared with the MSG group. The magnitude of this modulatory effect exceeded that observed in the ATX-treated group, supporting the superior capacity of GBB to attenuate apoptosis- and inflammation-related neurotoxicity (Fig. 8). MSG-induced neuroinflammation was evidenced by the marked upregulation of MMP9 at both the transcript and protein levels, accompanied by a substantial reduction in SOCS3 and JAK-2 protein expression. GBB treatment attenuated these inflammatory alterations, as demonstrated by 11.67-fold and 1.40-fold reductions in MMP9 mRNA and protein levels, respectively, compared to the MSG group. In parallel, GBB significantly increased SOCS3 and JAK-2 protein expression by 2.40- and 3.80-fold, respectively, indicating the restoration of inflammatory regulatory signaling and a stronger modulatory effect compared with the MSG-induced pathological state (Fig. 9). The mRNA expression levels of Beclin-1 and β-catenin were significantly downregulated in the MSG-treated group by 70% (*F*(3,21) = 143.3, *P* < 0.0001) and 55.7% (*F*(3,21) = 990.4, *P* < 0.0001), respectively, compared with the control group. GBB treatment effectively counteracted this suppression, significantly restoring both Beclin-1 and β-catenin expression relative to the MSG group. These findings suggest that GBB preserves autophagy-associated signaling and Wnt/β-catenin pathway activity under MSG-induced neurotoxicity conditions (Fig. 10).

## Discussion

Microbial biopriming, the pre-germination inoculation of seeds with beneficial microorganisms, represents a seed-level biotechnological strategy for modulating cereal metabolism and enhancing functional bioactivity. In this study, microbial biopriming redirected the metabolic and neurofunctional profiles of germinated barley in a strain-dependent manner. *B. subtilis* biopriming produced a phenolic- and B-vitamin-enriched profile, whereas *A. niger* preferentially enhanced amino acid accumulation, indicating distinct microbial effects on the metabolic reprogramming of barley. Among the tested extract preparations, the *B. subtilis* bioprimed extract exhibited the most pronounced neuroprotective efficacy in the MSG-induced ADHD-like rat model, as reflected by improved behavioral outcomes, restoration of neurotransmitter homeostasis, attenuation of oxidative stress, calcium dysregulation, neuroinflammatory signaling, and apoptosis-associated molecular alterations, together with modulation of autophagy- and Wnt/β-catenin-related pathways. These findings provide a framework for discussing the strain-specific metabolic remodeling of germinated barley, its functional antioxidant and neurobehavioral relevance, the molecular mechanisms underlying GBB-mediated neuroprotection, and the translational potential of microbial biopriming in developing cereal-derived functional food ingredients.

The phytochemical and nutritional differences observed among the bioprimed extracts reflect the distinct mechanisms by which each microorganism modifies the cereal matrix during germination. Phenolic enrichment in GBB can be attributed to microbial elicitation of phenylpropanoid metabolism through phytohormonal signaling, enzyme induction, and regulation of flavonoid-associated miRNAs^47,48^. This is consistent with reported increases in phenolic content following *B. subtilis* fermentation of cornmeal and soybean^49^, potentially mediated by bacterial carbohydrase-driven disruption of cell wall structures and liberation of bound phenolics^50^. Despite this enrichment, GBB exhibited moderate antioxidant activity, which may reflect *the B. subtilis*-mediated detoxification of phenolic acids via the padC/PadR-regulated phenolic acid decarboxylase system, which converts phenolic acids into lower-molecular-weight vinyl derivatives with diminished redox activity^51^. In contrast, the reduced total phenolic content of GBF likely reflects fungal-mediated transformation of phenolic constituents through hydroxylation, demethylation, dehydrogenation, glycoside hydrolysis, aromatic ring cleavage, and β-glucosidase- or esterase-mediated conversion into smaller, more polar derivatives^52,53^, a pattern consistent with observed tea polyphenol degradation during *A. niger* growth^54^. The superior antioxidant performance of GBF may therefore be attributed to the enzymatic liberation of bound phenolics into bioavailable antioxidant metabolites^55–57^, compounded by its nearly twofold higher protein content, consistent with the established role of *A. niger* in protein biosynthesis and cereal fortification^58,59^. Amino acid and FTIR analyses further corroborated the fungal enhancement of protein-derived metabolites in germinated barley.

Differential B-vitamin profiles across the extracts reflected the strain-specific metabolic potential of microbial treatments. The enrichment of GBF in vitamins B1 and B6 is consistent with fungal thiamine and pyridoxine biosynthesis mediated by *thiA* and *nmt1*^60,61^. In contrast, GBB markedly elevated riboflavin (B2; ~53-fold), folate (B9; ~3.9-fold), and cobalamin (B12; ~13-fold) relative to GBE, in accordance with the well-documented biosynthetic capacity of *Bacillus* spp. for B-complex vitamins^62–64^. Riboflavin (vitamin B2) biosynthesis in *B. subtilis* is governed by the *rib* operon (*ribA*, *ribB*, *ribG*, *ribH*, *ribT*)^65–67^, while folate (vitamin B9) biosynthesis proceeds through *folE*, *folB*, *folK*, *folP*, and *folC*^68,69^. Particularly striking is the 13-fold cobalamin (vitamin B12) accumulation in GBB, a finding of considerable nutritional significance, as B12 is synthesized exclusively by select bacteria and archaea, with no equivalent biosynthetic capacity in plants, fungi, or animals^70^, rendering cereal-derived matrices inherently devoid of this micronutrient. This enrichment suggests that *B. subtilis* biopriming can confer measurable cobalamin biofortification to an otherwise B12-null food matrix. Although *B. subtilis* is not a prolific natural cobalamin producer, its Generally Recognized As Safe (GRAS) status, well-characterized genetics, and genome-encoded cobalamin riboswitches and regulatory elements involved in cobalamin-dependent metabolism^71,72^. These strain-specific metabolic shifts are consistent with previous evidence showing that microbial- and processing-based interventions can substantially reshape barley phytochemistry and functional quality. Prior barley-focused studies have corroborated the modulatory potential of microbial and bioprocessing interventions on grain phytochemistry. *B. subtilis* fermentation of highland barley bran enhanced polyphenol release and antioxidant activity through the conversion of bound phenolics into extractable forms^30,73^, while *A. niger* 4Q alone or combined with *Cyberlindnera fabianii* J2 improved the antioxidant and flavor properties of whole-grain barley^74^. *Aspergillus oryzae* fermentation similarly elevated phenolic content and antioxidant capacity^75^, lactic acid bacteria fermentation of sprouted barley increased free amino acids and γ-aminobutyric acid while reducing phytic acid^76^, and ultrasonication-assisted germination enhanced phenolics, flavonoids, and antioxidant activity in black highland barley^77,78^. Microbial and physical bioprocessing can substantially remodel the nutritional and functional landscape of barley. Most strategies operate post-germination or through physical pretreatment. This study applied biopriming as an upstream seed-level intervention, positioning microorganisms to interact during germination, potentially driving a distinct phytochemical fingerprint through microbial enzymatic activity and seed metabolism.

**Fig. 11:**
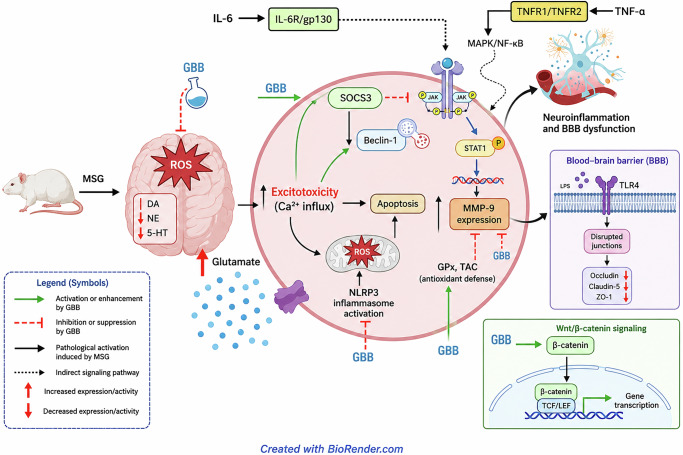
Proposed mechanism underlying the neuroprotective effects of GBB in the MSG-induced ADHD-like rat model. MSG induces oxidative stress, glutamate-mediated excitotoxicity, mitochondrial dysfunction, NLRP3 inflammasome activation, apoptosis, neuroinflammation, and BBB dysfunction, leading to impaired neurotransmitter homeostasis. GBB mitigates these pathological events by suppressing reactive oxygen species (ROS) generation, enhancing antioxidant defenses (GPx and TAC), attenuating NLRP3-mediated inflammatory signaling and apoptosis, reducing MMP-9 expression, modulating the IL-6/JAK/STAT/SOCS3 signaling axis, and promoting Wnt/β-catenin signaling, thereby contributing to neuroprotection. Solid green arrows indicate activation or enhancement by GBB; red dashed lines indicate inhibition or suppression by GBB; solid black arrows represent pathological signaling induced by MSG; dotted arrows denote indirect signaling interactions; upward (↑) and downward (↓) arrows indicate increased and decreased expression or activity, respectively. Created with BioRender.com.

The compositional advantages conferred by *B. subtilis* biopriming, particularly the enrichment of neuroprotective polyphenols and B-complex vitamins, were reflected in the superior in vivo performance of GBB, as demonstrated in an MSG-induced ADHD-like rat model. GBB improves behavioral performance, restores DA, NE, and 5-HT levels, and reduces glutamate accumulation, indicating the modulation of monoaminergic and excitatory neurotransmission^79,80^. These effects were accompanied by the restoration of Beclin-1 and suppression of apoptotic markers and cathepsin D, suggesting improved autophagic balance and reduced neuronal injury. Given that dysregulated autophagy, apoptosis, and neuroinflammation contribute to altered brain development and ADHD-related dysfunction^81,82^, the protective effects of GBB may be partly attributed to its polyphenol-rich profile, as polyphenols can promote autophagy, facilitate the clearance of misfolded proteins, and attenuate oxidative and inflammatory injury^83^. GBB also reduced NLRP3 inflammasome activation, a pathway implicated in MSG-induced caspase-1 activation, cytokine release, DA turnover, and striatal dopaminergic degeneration^84–86^. This effect is consistent with the anti-inflammatory and neuroprotective properties of natural polyphenols^87^ and may be further supported by increased SOCS3 levels, which restrain cytokine-mediated JAK/STAT inflammatory signaling^88^. In addition, GBB downregulated MMP9, a key mediator of neuroinflammation and blood–brain barrier (BBB) disruption. Excessive MMP9 activity compromises tight junctions and pericyte integrity, promotes cytokine infiltration, and contributes to neuronal apoptosis and death^89,90^. Elevated MMP9 levels have been associated with ADHD symptom severity and cognitive impairment^91^. Therefore, the marked reduction in MMP9 mRNA and protein levels after GBB treatment suggests the preservation of BBB integrity and attenuation of inflammatory neuronal injury. Concurrently, GBB upregulated β-catenin levels, indicating activation of the Wnt/β-catenin signaling pathway, which is essential for neuronal development, synaptic plasticity, and cognitive function^92^ (Fig. 11). These molecular effects can be mechanistically linked to the enrichment of syringic acid and rutin, both of which possess neuroprotective, antioxidant, and cytoprotective activities^93–95^. Elevated levels of vitamins B2 and B12 may further support neuronal energy metabolism, myelin integrity, DNA synthesis, and neurofunctional recovery^96–98^. Immune dysregulation, oxidative stress, and micronutrient imbalance have been increasingly implicated in neurodevelopmental and neurodegenerative disorders^99,100^. In ADHD, clinical evidence suggests that micronutrient deficiencies contribute to the disease pathophysiology^59^. Reduced vitamin B12 levels have been reported in children with ADHD and are associated with psychosomatic symptoms and learning difficulties^101^, whereas lower levels of vitamins B2, B6, and B9 have been observed in adults and are correlated with diagnosis and symptom severity^102^. Therefore, the pronounced enrichment of B vitamins in GBB may partially explain its superior behavioral, biochemical, and molecular effects on the brain. Mechanistically, these vitamins support mitochondrial energy metabolism, one-carbon metabolism, neurotransmitter synthesis, myelin maintenance, and redox homeostasis. Riboflavin (B2), through its coenzyme forms flavin mononucleotide and flavin adenine dinucleotide, contributes to oxidative metabolism and antioxidant defense^103,104^, while folate (B9) and cobalamin (B12) regulate DNA methylation, homocysteine remethylation, nucleotide synthesis, and neuronal development, which are essential for synaptic function and cognitive performance^105^. In ADHD animal models, neuroinflammation, impaired autophagy, and oxidative stress compromise BBB integrity in the prefrontal cortex and hippocampus, regions essential for attention, impulsivity control, memory, and executive function^82,106–109^. Barley-derived phenolic acids and flavonoids may counteract these effects by reducing lipid peroxidation, inflammatory signaling, and neuronal vulnerability, whereas polyphenols may modulate autophagy and apoptosis through AMPK/mTOR- and SIRT1-related pathways. Barley-derived phenolics may attenuate lipid peroxidation, inflammatory activation, autophagy dysfunction, and apoptosis through AMPK/mTOR- and SIRT1-related pathways^2,83^. Accordingly, the combined enrichment of B vitamins and phenolic metabolites in GBB may explain its protective effects in the MSG-induced ADHD-like model by coordinating oxidative, inflammatory, apoptotic, autophagic, and neurotransmitter-related responses.

In conclusion, this study demonstrates that *B. subtilis* biopriming enhances the phytochemical, antioxidant, and neuroprotective properties of germinated barley, with GBB exhibiting the greatest biological activity among the investigated extracts. To the best of our knowledge, this is the first report to integrate microbial seed biopriming with metabolite profiling, comparative B-vitamin profiling, antioxidant evaluation, and mechanistic neuroprotective validation in an MSG-induced ADHD-like rat model. Unlike conventional fermentation or physically assisted germination, microbial biopriming acts during the metabolically active germination phase, promoting phenolic acid accumulation together with strain-dependent modulation of the comparative B-vitamin profile. The distinctive phytochemical and comparative B-vitamin profiles of GBB are associated with improved behavioral performance, restoration of monoaminergic and glutamatergic homeostasis, attenuation of oxidative stress, and modulation of neuroinflammatory, apoptotic, autophagic, NLRP3, MMP-9, and Wnt/β-catenin-related pathways. These findings support the potential of *B. subtilis* biopriming as an effective strategy for developing functionally enhanced cereal-based foods with neuroprotective potential. Nevertheless, further investigations are required to substantiate the mechanistic relationships underlying the observed neuroprotective effects, characterize the bioavailability of the identified bioactive constituents, and assess the translational relevance of these findings within the clinical and dietary intervention frameworks. Several methodological considerations should be acknowledged when interpreting the present findings and designing future studies. The B-vitamin analysis was primarily intended to provide comparative profiling across the investigated extracts; therefore, the reported values should be interpreted as indicators of relative enrichment rather than as definitive absolute concentrations. However, the use of authentic reference standards, harmonized chromatographic conditions, and limit-of-quantification-based acceptance criteria strengthened the analytical consistency, reproducibility, and internal comparability of the data generated. Furthermore, although the MSG-induced ADHD-like rat model provides a valuable experimental platform for evaluating neurobehavioral, biochemical, and molecular perturbations, it does not fully reproduce the etiological heterogeneity and clinical complexity of human ADHD, thereby limiting direct translational extrapolations. Future investigations should incorporate comprehensive multi-omics approaches, extend metabolomic coverage to non-polar and lipid-derived constituents, evaluate microbial persistence and metabolic activity throughout germination, and refine biopriming parameters across diverse microbial strains and cereal matrices. Further validation using complementary neurobehavioral models, extended intervention protocols, pharmacokinetic and bioavailability assessments, and rigorously designed clinical trials will be essential to establish the functional relevance and translational potential of microbially bioprimed barley.

## Methods

### Grain preparation and biopriming treatments

Barley grains (*H. vulgare* L., Gramineae) were obtained from the Agricultural Research Center, Giza, Egypt, and authenticated at the Agricultural Museum, Dokki, Giza, Egypt. A voucher specimen (No. 1482) was deposited at the Herbarium of the Flora and Phytotaxonomy Research Department (CAIM), Horticultural Research Institute, Agricultural Research Center, Giza, Egypt. The grains were manually sorted to remove damaged seeds, sieved to eliminate dust and fine particles, and stored at 4 °C until use. For the experiment, grains were divided into three 500 g portions: untreated control, *B. subtilis* bioprimed grains, and *A. niger* bioprimed grains. Before biopriming, all samples were surface-sterilized in 70% ethanol for 10 min and rinsed three times with sterile, distilled water. A *B. subtilis* isolate, strain 10^−4^, obtained from the Faculty of Pharmacy, Cairo University, was cultured on Sabouraud dextrose agar (SDA; Research Lab Fine Chem Industries, Mumbai, India) at 37 °C for 3–4 days. The bacterial suspension was adjusted to 10⁸ CFU ml⁻¹ using the 0.5 McFarland standard and monitored at 600 nm using a UV–Vis spectrophotometer (PGT80, Lutterworth, UK), and then diluted with sterile distilled water to 10⁵ CFU ml⁻¹^110^. Surface-sterilized barley grains were immersed in the bacterial suspension for 1 h under aseptic conditions, air-dried, and then subjected to germination^111^. *A. niger* ATCC 10404 was obtained from the Microbiological Resources Center (MIRCEN) of the Faculty of Agriculture, Ain Shams University. The fungus was cultured in 250 ml Erlenmeyer flasks containing 100 ml potato dextrose broth (PDB) at 25 ± 2 °C for 14 days to promote sporulation^112^. Spores were harvested by filtration through sterile gauze, and the suspension was adjusted to 10⁶ spores ml⁻¹ using a hemocytometer. Surface-sterilized barley grains were soaked in a freshly prepared spore suspension for 12 h under aseptic conditions, briefly rinsed with sterile distilled water to remove excess inoculum, and then subjected to germination^113,114^.

### Germination and extract processing

Following biopriming, all seed groups were immersed in sterile distilled water for 12 h to standardize hydration. Germination was carried out by spreading grains on a sterile moistened cloth and incubating them in the dark at room temperature for four days, with periodic water sprinkling to maintain adequate moisture. Germinated grains were air-dried to a constant weight, ground, and macerated in distilled water (500 ml) for 15 days. The aqueous extracts were collected, decanted after 24 h, and the supernatants were concentrated under reduced pressure using a rotary evaporator (Rotavapor® R-100, Büchi, Switzerland). The extraction yield was expressed as a percentage weight/weight (% *w*/*w*) relative to the dry weight of the starting material and was calculated as follows:

$${\rm{Yield}}\left( \% \right)=[{\rm{Weight\; of\; dried\; extract}}\left({\rm{g}}\right)/{\rm{Weight\; of\; dry\; starting\; material}}\left({\rm{g}}\right)]\times 100$$

### Phytochemical analysis

The total phenolic content of GBE, GBB, and GBF was determined using the Folin–Ciocalteu method^115^. Briefly, 125 μl of each extract was mixed with 500 μl deionized water and 125 μl Folin–Ciocalteu reagent, and allowed to react for 6 min at room temperature. Subsequently, 1.25 ml of 7% sodium carbonate solution and 1 ml deionized water were added to a final volume of 3 ml. The mixture was incubated in the dark for 1.5 h at room temperature, and the absorbance was measured at 760 nm using a UV–Vis spectrophotometer (Shimadzu UV-2600, Kyoto, Japan). Quantification was performed using gallic acid as the standard over 1–20 µg ml⁻¹, and the results were expressed as gallic acid equivalents (GAE) per 100 g of extract using the calibration equation *y* = 0.0047*x* − 0.013 (*R*² = 0.9958). The total flavonoid content was determined using the aluminum chloride colorimetric method^116^, with quercetin as the standard. Briefly, 5 ml of each sample was mixed with 10 ml ethanol, 1 ml of 1 M potassium acetate, 1 ml of 10% aluminum chloride, and 3 ml distilled water. The mixture was vortexed, incubated in the dark for 30 min at room temperature, and the absorbance was measured at 415 nm^117^. The flavonoid content was calculated from a quercetin calibration curve using the equation *y* = 0.0052*x* + 0.1069 (*R*² = 0.999), as described by Garuba et al.^114^, and expressed as mg quercetin equivalents (QE) per 100 g of sample. The protein content was determined using the Kjeldahl method^115^. Briefly, 1 g of each sample was digested with concentrated sulfuric acid using a potassium–copper sulfate catalyst. After neutralization with sodium hydroxide, the liberated ammonia was distilled and titrated with standard hydrochloric acid. Total nitrogen was calculated from the titration values and converted to crude protein using a 6.25 factor.

### HPLC analysis

HPLC analyses of phenolics, amino acids, and B vitamins in GBE, GBB, and GBF were performed using an Agilent 1260 series system. Phenolic constituents were analyzed on a Zorbax Eclipse Plus C8 column (4.6 × 250 mm, 5 μm) maintained at 40 °C, using water (A) and 0.05% trifluoroacetic acid in acetonitrile (B) at 0.9 ml min⁻¹ with a linear gradient of 82% A at 0–1 min, 75% A at 11 min, 60% A at 18 min, and re-equilibration to 82% A from 22 to 24 min; the injection volume was 5 μl, and detection was performed at 180–480 nm. Phenolic compounds were identified by comparing retention times and UV spectra with authentic standards and quantified using external calibration curves prepared from serial standard dilutions of 1–75 µg ml⁻¹, which showed excellent linearity (*R*² > 0.999; Fig. S5). The LOD and LOQ were calculated at signal-to-noise ratios of 3 and 10, respectively, and the calibration equations, LOD, LOQ, and linearity values are presented in Table 1 (Fig. S5 and Supplementary File). Amino acids were analyzed on an Eclipse Plus C^18^ column (4.6 × 250 mm, 5 μm) using sodium phosphate dibasic/sodium borate buffer, pH 8.2 (A), and acetonitrile:methanol:water (45:45:10, *v*/*v*/*v*) (B) at 1.5 ml min⁻¹, with a gradient of 98% A at 0–0.84 min, 43% A at 33.4 min, 0% A at 39.3 min, and 98% A at 40 min. Detection was performed using DAD at 338 nm and fluorescence detection at 340/450 nm from 0 to 27 min and 266/306 nm from 27 to 35 min, with a wavelength transition after lysine elution to monitor FMOC-derivatized amino acids. Amino acids were quantified using external calibration curves generated from authentic standards of 10–250 nmol ml⁻¹ subjected to the same ortho-phthalaldehyde/9-fluorenylmethyl chloroformate (OPA/FMOC) derivatization and chromatographic conditions as the samples (Figure S6); the LOQ was 10 nmol ml⁻¹, defined as the lowest concentration producing a signal-to-noise ratio ≥ 10 under the optimized conditions. B vitamins were analyzed on a ZORBAX SB-C8 column (4.6 × 150 mm, 5 μm) using water containing 0.01% trifluoroacetic acid, pH 2.9 (A), and methanol (B) at 1.5 ml min⁻¹ in gradient mode, with a 5 μl injection volume and detection at 270 nm to improve sensitivity for mixed water-soluble B vitamins. The peaks were identified by comparison with authentic standards (Fig. S7), and vitamin concentrations were estimated using single-point external calibration at 5 µg ml⁻¹ to support comparative profiling among extracts under identical chromatographic conditions (Table 4). The baseline noise (σ = 10 mAU·s) was determined from a stable segment adjacent to each analyte peak using Agilent OpenLab CDS, and the LOQ was estimated according to ICH Q2(R2) using the equation LOQ = 10σC_std_/Area_std_. where σ is the baseline noise, C_std_ is the concentration of the standard, and Area_std_ is the peak area of the corresponding standard. The calculated LOQs ranged from 0.51 to 2.30 µg/ml, confirming the adequate sensitivity of the developed method for B-vitamin determination. All measurements were performed in triplicate, and the results are expressed as the mean ± SEM.

**Table 4:** Calculated limits of quantitation (LOQ) for B-vitamins determined by HPLC-DAD using a single-point calibration (5 µg ml⁻¹)

Vitamin	Retention time (min)	Peak area (mAU·s)	LOQ (µg ml⁻¹)
B₁ (Thiamine)	1.20	217.19	2.30
B₆ (Pyridoxine)	1.82	404.11	1.24
B₃ (Niacin)	2.13	284.38	1.76
B₉ (Folic acid)	3.50	371.79	1.34
B₂ (Riboflavin)	3.78	831.41	0.60
B₁₂ (Cobalamin)	3.99	986.78	0.51

Baseline noise (σ = 10 mAU·s) was measured in a flat region adjacent to each peak, and LOQ values were derived according to the ICH Q2(R2) guideline (LOQ = 10σC_st_d/Area_st_d).

### Fourier transform infrared (FT-IR) spectrometry

FT-IR spectra of GBE, GBB, and GBF were recorded using a Shimadzu IR spectrophotometer by the KBr disc method^118^. Analysis was performed to evaluate the treatment-induced changes in the functional groups and chemical profiles of the barley extracts.

### In vitro antioxidant activity

The 2,2-diphenyl-1-picrylhydrazyl (DPPH) radical scavenging activity was evaluated according to ref.^119^ with slight modifications. Briefly, 2.4 ml of freshly prepared 0.1 mM DPPH solution in methanol was mixed with 1.6 ml of each extract at different concentrations (12.5–150 μg ml⁻¹). The mixture was vortexed and incubated in the dark at room temperature for 30 min. Absorbance was measured at 517 nm using a UV–Vis spectrophotometer (Shimadzu UV-2600, Kyoto, Japan). The DPPH scavenging activity was calculated using the following equation:

$${\rm{DPPH\; radical\; scavenging\; activity}}\left( \% \right)=[\left({{\rm{A}}}_{0}-{{\rm{A}}}_{1}\right)/{{\rm{A}}}_{0}]* 100$$

Where *A*_*0*_ is the absorbance of the control (DPPH solution without extract) and *A*_*1*_ is the absorbance of the sample solution.

### Functional evaluation of bioprimed extracts

To support the bioactivity of phytochemical-enhanced barley extracts, an in vivo assay was conducted using a MSG-induced ADHD-like behavioral model in rats. Rats were selected because they provide a well-established experimental platform for evaluating ADHD-related neurobehavioral, neurochemical, biochemical, and molecular alterations under controlled conditions. Rodent models are widely used in ADHD research to assess hyperactivity, attention-related behavior, learning and memory deficits, neurotransmitter imbalance, oxidative stress, neuroinflammation, apoptosis, autophagy, and brain signaling pathways^120–122^. MSG exposure has been reported to induce ADHD-like behavioral abnormalities in rats, accompanied by oxidative stress, inflammatory activation, neurotransmitter disturbance, apoptosis, and dysregulated neuroprotective signaling^32,123^. Therefore, this model was considered appropriate for evaluating the neuroprotective potential of different extracts and their effects on behavioral and molecular outcomes. Sixty male Western albino rats aged 4–6 weeks and weighing 25–40 g were obtained from the Nile Company for Pharmaceuticals and Chemical Industries (Cairo, Egypt). Animals were housed under controlled conditions at 25 °C, 55% relative humidity, and a 12 h light/dark cycle with free access to a standard diet and water. All procedures were conducted in accordance with the *Guide for the Care and Use of Laboratory Animals* (National Institutes of Health, 1996) and approved by the Research Ethics Committee, Faculty of Pharmacy, October 6 University, Giza, Egypt (Approval No. PME-PH-2503001). This study was reported in accordance with the ARRIVE guidelines. The rats were randomly assigned to six groups, with ten rats per group. Following behavioral assessments, the rats were anesthetized with 3% isoflurane for 10 min, and adequate anesthesia depth was confirmed before euthanasia by cervical dislocation. Brain tissues were immediately excised and processed for biochemical, molecular, and histopathological analysis. For the selected endpoints, analyses were performed using *n* = 6 per group because one sample was unsuitable for analysis. The sample sizes for each endpoint are indicated in the corresponding figure legends, and statistical analyses were performed using the valid sample number. The experimental groups were treated as follows for 60 consecutive days: Group I (Control) received distilled water only; Group II (ADHD model) received MSG (400 mg/kg/day, oral gavage)^124^; Group III (ADHD + GBE) received MSG (400 mg/kg/day) co-administered with GBE (300 mg/kg/day)^125^; Group IV (ADHD + GBB) received MSG (400 mg/kg/day) co-administered with GBB (300 mg/kg/day); Group V (ADHD + GBF) received MSG (400 mg/kg/day) co-administered with GBF (300 mg/kg/day); and Group VI (ADHD + ATX) received MSG (400 mg/kg/day) co-administered with ATX (1 mg/kg/day)^126^.

### Behavioral experiments

Behavioral assessments were conducted during the final two days of the experiment. Cognitive function and exploratory activity were evaluated using Y-maze and open-field tests. Spontaneous alternation behavior may indicate spatial working memory, a form of short-term memory^127^. A black wood Y-maze, including a symmetrical triangular core space and three arms designated A, B, and C, was used as previously detailed^128^. Spontaneous alternation behavior was assessed by sequential entry into overlapping triplet arm combinations (e.g., CAB and ABC) in a Y-maze. Rats were placed at the entrance of one arm and allowed to navigate freely for 8 min, and entries were recorded when the hind paws were fully within an arm. The SAP was calculated as follows: SAP (%) = [number of alternations/(total arm entries − 2)] × 100. Locomotor activity was assessed in a black rectangular open-field box (72 × 72 × 36 cm) with a floor subdivided into 16 equal squares (18 × 18 cm), as previously described^128^. The rats were placed individually in the center and allowed to explore freely for 3 min. The apparatus was cleaned with 70% ethanol between trials. The measured parameters included ambulation (lines crossed), rearing frequency, total mobile time, and mean speed (distance/time).

### Histopathology

Following behavioral assessments, the rats were anesthetized with 3% isoflurane until a surgical plane of anesthesia was achieved. Adequate anesthesia depth was confirmed prior to euthanasia by cervical dislocation. Brain tissues were immediately excised, rinsed in ice-cold saline, and allocated as follows: seven brains per group were designated for biochemical and molecular analyses, of which 100 mg was isolated for RNA extraction, and the remainder was homogenized in phosphate-buffered saline (PBS; pH 7.4, 10% *w*/*v*); three brains per group were fixed in 10% neutral-buffered formalin for histopathological examination. Fixed tissues were routinely processed, paraffin-embedded, sectioned at 4 µm, and stained with hematoxylin and eosin (H&E) for light microscopy. All procedures were conducted in strict compliance with the Research Ethics Committee of the Faculty of Pharmacy, October 6 University (Permit No. PME-PH-2503001), and the NIH Guide for the Care and Use of Laboratory Animals (1996).

### Biochemical evaluations

The in vivo study followed a staged comparative design. GBE, GBB, and GBF were initially evaluated for phytochemical, antioxidant, behavioral, neurochemical, oxidative stress-related, and histopathological effects in the MSG-induced ADHD-like rat model. Based on this comparative assessment, GBB exhibited the most pronounced overall bioactivity and was selected for subsequent mechanistic analyses targeting apoptosis, autophagy, NLRP3 inflammasome activation, MMP9, SOCS3/JAK/STAT signaling, and Wnt/β-catenin modulation. Biochemical analyses were performed on 10% brain-tissue homogenates. Total antioxidant capacity (TAC; Cat. No. TA2513), and glutathione peroxidase activity (GPx; Cat. No. GP2524; Bio Diagnostic, Giza, Egypt) were measured according to the manufacturer’s instructions. Calcium levels (Ca²⁺; Cat. No. CAL 103060 (Bio Diagnostic, Giza, Egypt) were determined using the same homogenate preparations. Brain NE, DA, 5-HT, and glutamate concentrations were quantified using ELK Biotechnology enzyme-linked immunosorbent assay (ELISA) kits (Cat. Nos. ELK8317, ELK8953, ELK8954, and ELK0812; Wuhan, China). β-Catenin, JAK2, SOCS3, and α-synuclein levels were measured using Sunlong Biotech kits (Cat. Nos. SL1294Ra, SL1398Ra, SL1122Ra, and SL1237Ra (Zhejiang, China), and NLRP3 and MMP9 were quantified using YL Biont kits (Cat. Nos. YLA1378RA, and YLA1065RA (Shanghai, China), according to the manufacturer’s protocols. Gene expression analysis was performed on RNA extracted using the QIAamp RNeasy Mini Kit (Qiagen, Germany). The expression of Bax, Bcl-2, β-actin, cathepsin D, Beclin-1, and MMP9 was analyzed using the Stratagene MX3005P real-time PCR system with the primer sequences listed in Table 5. Relative expression was calculated using the 2^−ΔΔCt^ method^129^.

**Table 5:** The sequence of the PCR primers

The target gene	The sequence
Bax	Forward 5’-CACCAGCTCTGAACAGATCATGA-3’ Reverse 5’-TCAGCCCATCTTCTTCCAGATGGT-3’
Bcl-2	Forward 5’-CACCCCTGGCATCTTCTCCTT-3’ Reverse 5’-AGCGTCTTCAGAGACAGCCAG-3’
Cathepsin D	Forward 5’-AGCACCTATGTGAAGAACGG-3’ Reverse 5’-TGGCTGCAACAAATACGAT-3’
Beclin-1	Forward 5’-TTGGCCAATAAGATGGGTCTGAA-3’ Reverse 5’-TGTCAGGGACTCCAGATACGAGTG-3’
MMP9	Forward 5’-TCGAAGGCGACCTCAAGTG-3’ Reverse 5’-TTCGGTGTAGCTTTGGATCCA-3’
β-actin	Forward 5’-TCCTCCTGAGCGCAAGTACTCT-3’ Reverse 5’-GCTCAGTAACAGTCCGCCTAGAA-3’

### Statistical analysis

Data were analyzed using GraphPad Prism® (version 9.5.0; GraphPad Software, San Diego, CA, USA). Group differences were assessed using one-way ANOVA followed by Tukey’s post hoc multiple comparisons test. Results are expressed as mean ± standard deviation (SD), with statistical significance set at *P* < 0.05.

## Supplementary information

Supplementary Information

## Data Availability

The original uncropped histological images supporting this study are publicly available in Figshare at: 10.6084/m9.figshare.31443124.

## Code availability

No custom code or software was generated or used during this study. Therefore, code availability is not applicable.
