# Supplementary material for: Microbial biopriming of germinated barley enhances phenolics and B-vitamins and improves neurobehavior in MSG-treated rats
Source: NPJ Sci Food. 2026 Jul 29;10:233. doi: 10.1038/s41538-026-01022-z (PMC13421475; doi:10.1038/s41538-026-01022-z)
Supplement: Supplementary file 1 — Supplementary Information [file 41538_2026_1022_MOESM1_ESM.docx]

**Supplementary File

Title: Microbial bio-priming of germinated barley enhances phenolics and B-vitamins and improves neurobehavior in MSG-induced rats**

**
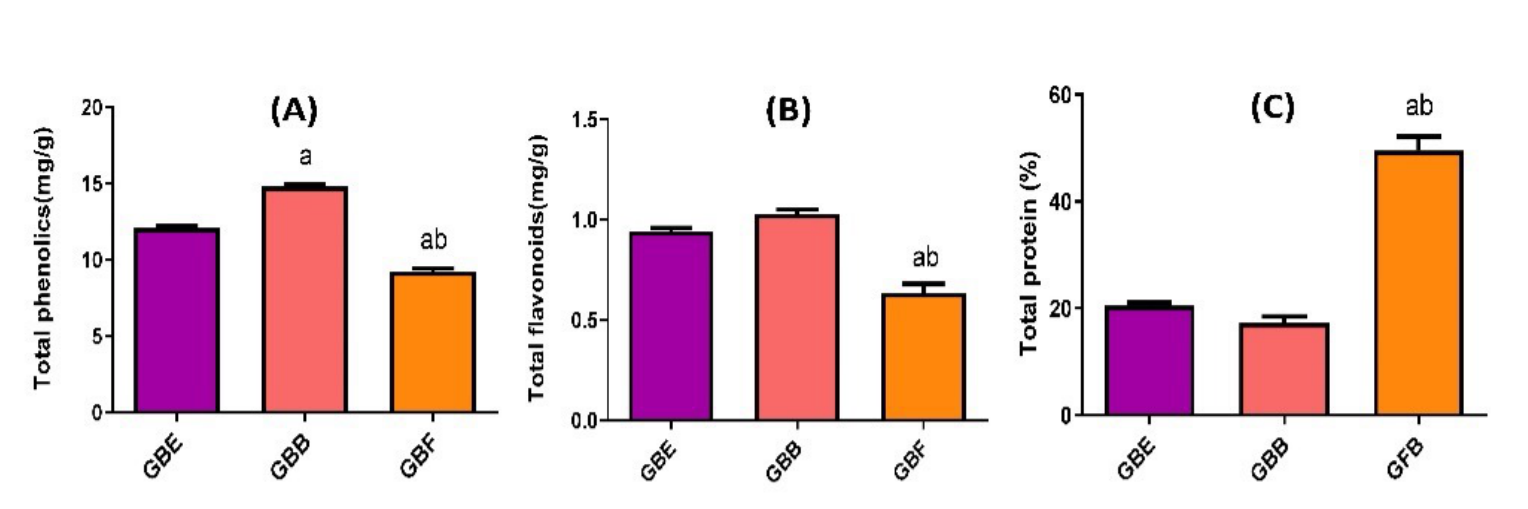
**

**Fig. S1.** Quantitative phytochemical characterization (A; total phenolic acid content, B; total flavonoid content, c; total protein content) of germinated barley (GBE), germinated barley bacterial (*Bacillus*) treated (GBB), and germinated barley fungal (*Aspergillus*) treated (GBF). Data presented as mean ± SEM. a, b: values considered significant compared to the GBE and GBB groups respectively—one-way ANOVA followed by Tukey’s as a post-hoc test presented P-value < 0.05.


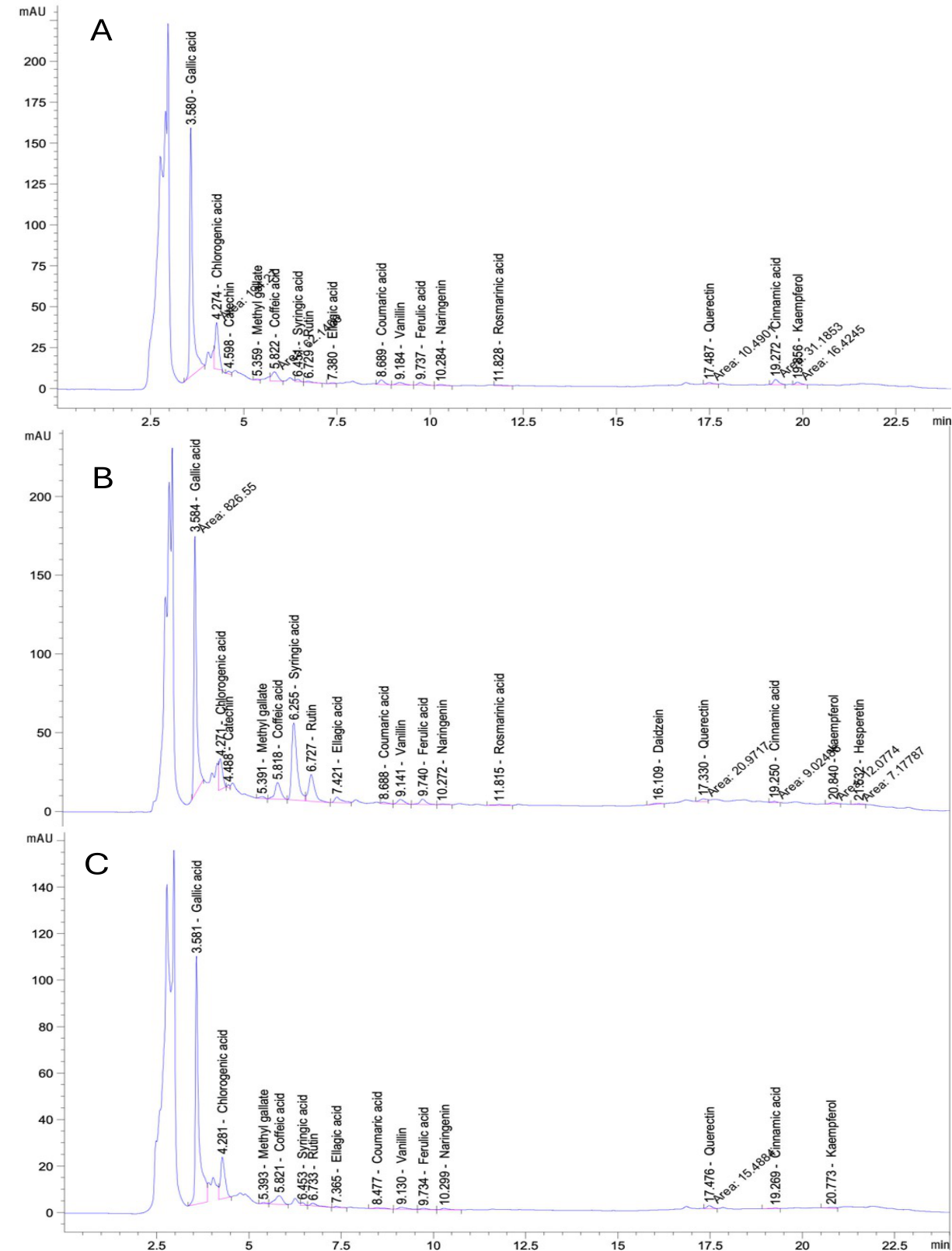


**Fig. S2.** High-performance liquid chromatography (HPLC) analysis of GBE; (A), GBB; (B), and GBF; (C), revealing their respective phenolic compound profiles.


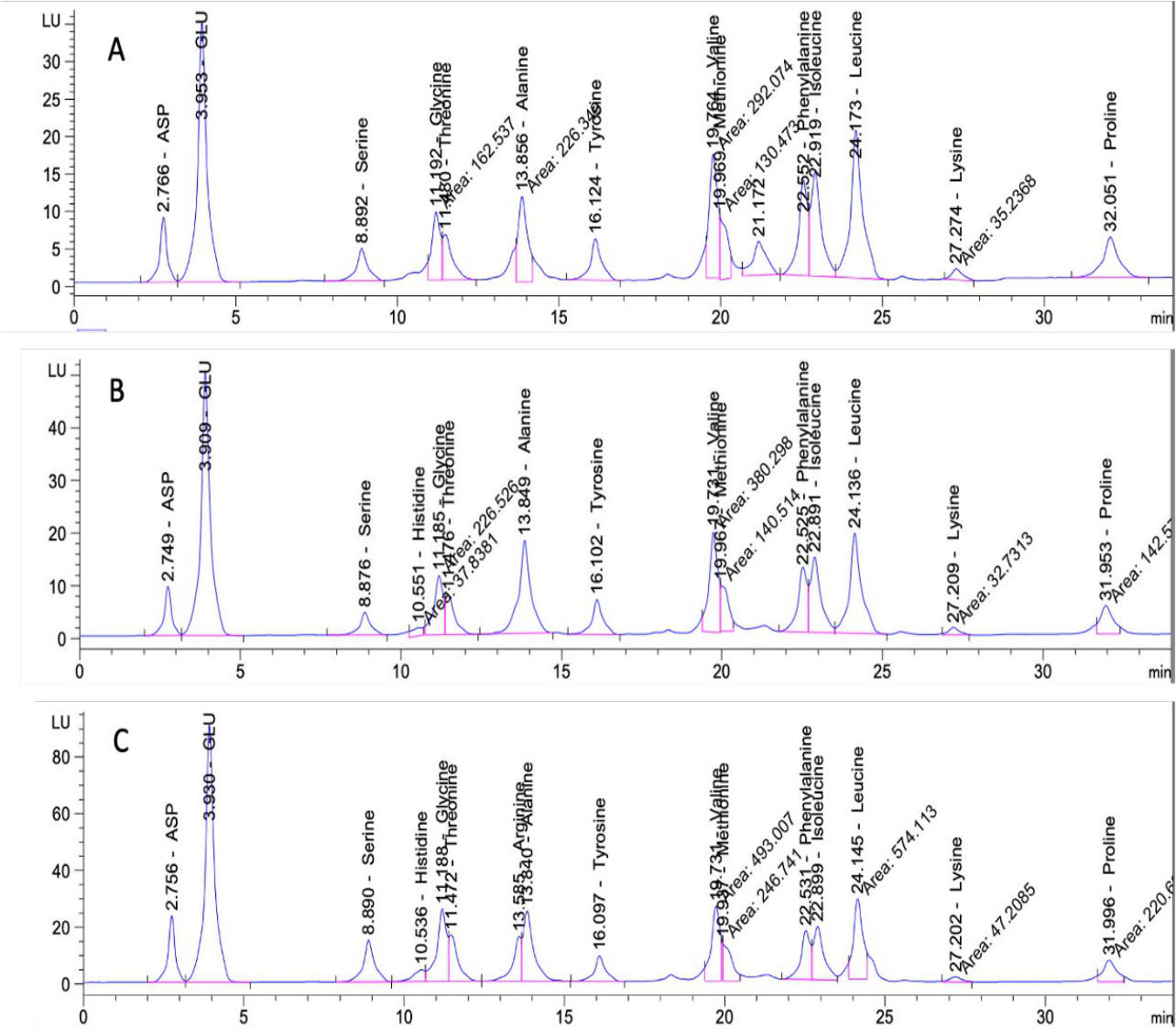


**Fig. S3.** High-performance liquid chromatography (HPLC) analysis of GBE; (A), GBB; (B), and GBF; (C), revealing their representive amino acid profiles.


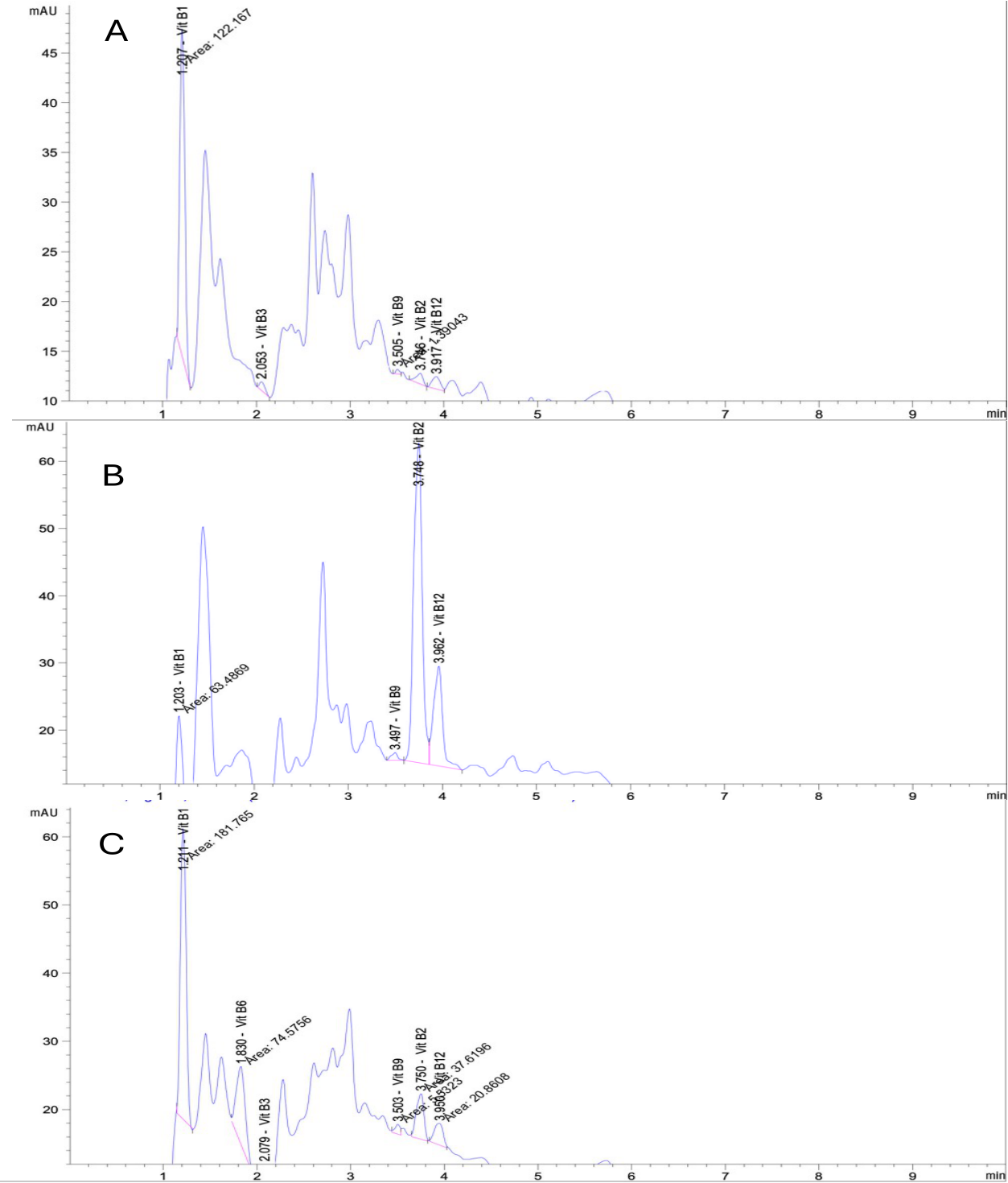


**Fig. S4.** High-performance liquid chromatography (HPLC) chromatograms of GBE; (A), GBB; (B), and GBF; (C), demonstrating the differential composition and distribution of B-vitamins across the three extracts.

**Fig. S5.** Calibration curves of standard phenolic and flavonoid compounds used for quantitative HPLC analysis. Each plot represents the linear correlation between concentration (µg/mL) and peak area (mAU) for individual standards. All compounds, including gallic acid, methyl gallate, ferulic acid, naringenin, chlorogenic acid, catechin, rosmarinic acid, daidzein, caffeic acid, syringic acid, quercetin, cinnamic acid, rutin, ellagic acid, kaempferol, hesperetin, coumaric acid, and vanillin, exhibited excellent linearity within the tested range (R² ≥ 0.999).

**Fig. S6.** Calibration curves of amino acid standards used for quantitative HPLC analysis after derivatization. Each plot illustrates the linear relationship between concentration (ng/mL) and peak area (mAU) for individual amino acids, including serine, histidine, valine, glycine, arginine, tyrosine, threonine, alanine, cystine, methionine, phenylalanine, isoleucine, leucine, lysine, and proline. All calibration curves exhibited excellent linearity (R² ≥ 0.997), confirming method accuracy and suitability for quantification.





**Fig. S7.** Representative HPLC chromatogram of standard B-group vitamins detected at 270 nm using a DAD detector. Peaks correspond to thiamine (vitamin B₁, Rt = 1.199 min), riboflavin (vitamin B₂, Rt = 3.780 min), niacin (vitamin B₃, Rt = 2.133 min), pyridoxine (vitamin B₆, Rt = 1.823 min), folic acid (vitamin B₉, Rt = 3.500 min), and cobalamin (vitamin B₁₂, Rt = 3.987 min). The chromatogram demonstrates clear resolution and reproducible retention times for each standard, confirming the method’s suitability for simultaneous quantification.
